# Evasion by Stealth: Inefficient Immune Activation Underlies Poor T Cell Response and Severe Disease in SARS-CoV-Infected Mice

**DOI:** 10.1371/journal.ppat.1000636

**Published:** 2009-10-23

**Authors:** Jincun Zhao, Jingxian Zhao, Nico Van Rooijen, Stanley Perlman

**Affiliations:** 1 Department of Microbiology, University of Iowa, Iowa City, Iowa, United States of America; 2 Institute for Tissue Transplantation and Immunology, Jinan University, Guangzhou, China; 3 Department of Molecular Cell Biology, Vrije Universiteit Medisch Centrum, Amsterdam, The Netherlands; University of Washington, United States of America

## Abstract

Severe Acute Respiratory Syndrome caused substantial morbidity and mortality during the 2002–2003 epidemic. Many of the features of the human disease are duplicated in BALB/c mice infected with a mouse-adapted version of the virus (MA15), which develop respiratory disease with high morbidity and mortality. Here, we show that severe disease is correlated with slow kinetics of virus clearance and delayed activation and transit of respiratory dendritic cells (rDC) to the draining lymph nodes (DLN) with a consequent deficient virus-specific T cell response. All of these defects are corrected when mice are treated with liposomes containing clodronate, which deplete alveolar macrophages (AM). Inhibitory AMs are believed to prevent the development of immune responses to environmental antigens and allergic responses by interacting with lung dendritic cells and T cells. The inhibitory effects of AM can also be nullified if mice or AMs are pretreated with poly I:C, which directly activate AMs and rDCs through toll-like receptors 3 (TLR3). Further, adoptive transfer of activated but not resting bone marrow–derived dendritic cells (BMDC) protect mice from lethal MA15 infection. These results may be relevant for SARS in humans, which is also characterized by prolonged virus persistence and delayed development of a SARS-CoV-specific immune response in individuals with severe disease.

## Author Summary

Severe Acute Respiratory Syndrome (SARS) occurred in human populations in 2002–2003 and was caused by a novel coronavirus (CoV). Human SARS was characterized by prolonged virus excretion, lymphopenia and delayed adaptive immune responses in patients with severe disease. Recently, small animal models have been developed that mimic some of the features of the human disease. Specifically, BALB/c mice infected with mouse-adapted SARS-CoV develop severe respiratory disease. Here, we show that the T cell response is defective in these mice and that this results from inefficient activation of the initial immune response to the virus. This defect can be corrected by several treatments, including depletion of inhibitory macrophages from the lungs and direct activation of respiratory dendritic cells, important in initiating the immune response or transfer of activated dendritic cells prior to infection. All of these modalities result in improved initiation of the immune response and an enhanced anti-virus T cell response. Inefficient activation of the immune response may play a role in human SARS, and our results suggest possible strategies that might be used to develop novel anti-viral therapies.

## Introduction

The lung is exposed to many challenges, both environmental and pathogenic. Defense of this portal must be tightly regulated so that appropriate immune responses to pathogens are mounted but responses to innocuous antigens are minimized. Alveolar macrophages (AM) play a central role in maintaining this immunological homeostasis [1],[2],[3]. In the lung, resident AMs are continuously encountering inhaled substances due to their exposed position in the alveolar lumen, but they are kept in a quiescent state. They function poorly as accessory cells for *in vitro* T cell activation [4],[5] and in many situations actively suppress the induction of adaptive immunity through their effects on alveolar and interstitial DCs and T cells [6],[7],[8]. *In vivo* elimination of alveolar macrophages using clodronate-filled liposomes (CL) leads to overt inflammatory reactions to otherwise harmless particulate and soluble antigens [9]. Alveolar macrophages adhere closely to alveolar epithelial cells (AECs) at the alveolar wall and are separated by a distance of only 0.2–0.5 µm from rDCs [6]. In macrophage-depleted mice, DCs have enhanced antigen-presenting function [6]. It has been estimated that the pool of murine alveolar macrophages can process up to 10^9^ intratracheally injected bacteria before there is “spillover” of bacteria to DCs and before adaptive immunity is induced [10].

Although the importance of such mechanisms to control undesirable responses to inert environmental antigens is self-evident, it is also axiomatic that countermeasures must be available to allow reversal of this inhibition after challenge with inhaled pathogenic (notably microbial) antigens. During infection with respiratory pathogens, such as influenza virus, antigen is acquired by respiratory dendritic cells (rDCs) and these cells must be sufficiently activated to overcome anti-inflammatory factors in the lungs. These rDCs then migrate to the lung draining lymph nodes (DLN) to initiate an antiviral CD8 T cell response [11],[12]. After the interaction of naive T cells with such antigen-bearing DCs, CD8 and likely CD4 T cells undergo activation and division in the DLNs and migrate into the lungs to eliminate virus-infected cells, leading to resolution of the infection [13],[14],[15]. Recently, a secondary peripheral interaction of CD8 T cells with antigen-bearing rDCs in the lung was found important for effective antiviral immunity [16]. Overall rDC activation is a prerequisite for initiation and maintenance of the immune response.

Patients with the Severe Acute Respiratory Syndrome (SARS), caused by a novel coronavirus (SARS-CoV), developed mild to fatal pulmonary disease, with a mortality incidence of 10% [17]. Patients with worse outcomes generally exhibited a more protracted clinical course, characterized by the development of Adult Respiratory Distress Syndrome (ARDS), as well as lymphopenia, neutrophilia and prolonged cytokine production [17],[18],[19],[20]. Virus could be detected in nasopharyngeal aspirate and feces for as long as 21 days after disease onset [19],[21]. Delayed virus clearance may have resulted from suboptimal T and B cell responses; suboptimal neutralizing antibody responses are detected in patients with severe disease [17],[18],[19],[20]. Numerous studies demonstrated that SARS-CoV infection fails to activate macrophages and dendritic cells. Although these cells can be infected, they are functionally impaired: antiviral cytokines such as type I interferon were not expressed and endocytic capacity (antigen capture) was compromised ([22],[23],[24],[25],[26],[27],[28] and reviewed in [29]). These unusual findings raised the possibility that initial infection with the virus resulted in delayed or suboptimal activation of the innate immune system. Inefficient activation of rDCs might be unable to counter the potent anti-inflammatory factors that are normally present in the lung, resulting in both a deficient T cell response and delayed kinetics of virus clearance.

Recently, rodent-adapted strains of SARS-CoV, which cause mild to fatal respiratory disease, were developed in several laboratories [30],[31]. Here, we demonstrate that lethal disease in mice infected with a mouse-adapted strain of SARS-CoV (MA15) can be prevented if AMs with anti-inflammatory properties are depleted from the lung prior to infection. Treatment with toll-like receptor (TLR) agonists to activate rDCs or transfer of activated bone marrow-derived dendritic cells (BMDC) also prevents a lethal outcome. Together, these results demonstrate that SARS-CoV, by inefficiently activating the immune system, uses a novel mechanism to evade immune recognition.

## Results

### AM depletion before inoculation protected BALB/c mice from lethal MA15 infection

SARS-CoV infection results in inefficient activation of macrophages and DCs *in vitro* [22],[23],[24],[25],[26],[27],[28] and slow virus clearance and a prolonged clinical course in humans [17],[18],[19]. Similarly, MA15 infection *in vitro* did not result in upregulation of CD86 on AM (Fig. S2, Gating shown in Fig. S1 A). To determine whether inhibitory AMs play a role in MA15-mediated severe lung disease, we depleted these cells by intranasal administration of clodronate liposomes (CL). CL are useful for depletion of AM, and to a lesser extent, alveolar/airway DCs [9], but intranasal administration does not affect the level of circulating macrophages [32]. As a control, we treated mice with PBS as described previously [33].

BALB/c mice were treated with 75 µl of CL or PBS intranasally (i.n.) and total lung cells were harvested after enzymatic digestion. After 24 h, there was a decrease of AMs (CD11c^+^CD11b^−^siglec F^+^ [34]) in the lung, both in frequency (>70%) and absolute number (from 5–6×10^4^ to 1–2×10^4^ cells/lung), in CL, but not PBS-treated mice (Fig. S3 A and B). By 48 h, approximately 90% of AMs in the lung were depleted (Fig. S3 A and B).

**Figure 1:**
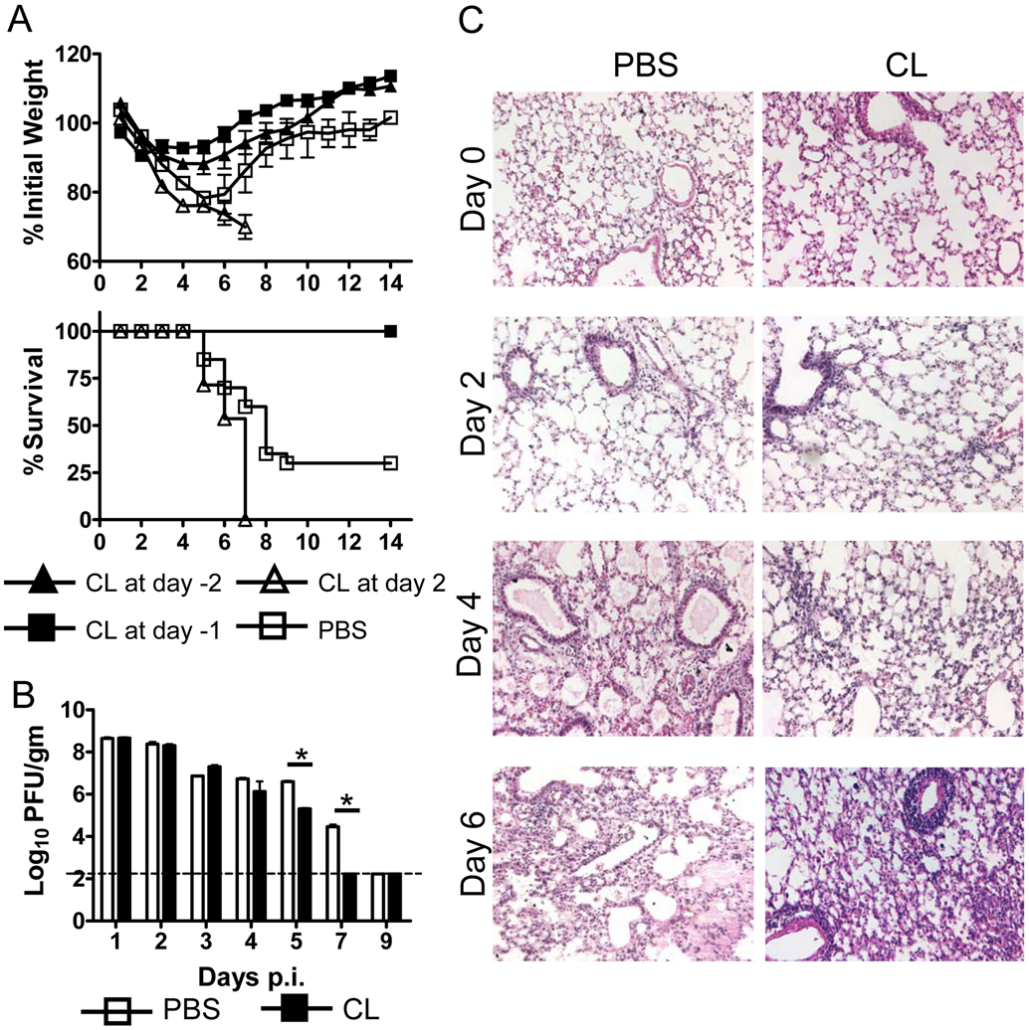
Effect of CL treatment on weight loss, mortality, histological changes and virus titers in MA15-infected BALB/c mice. (A) BALB/c mice (6–8 weeks old) were treated with 75 µl CL or PBS at before or after intranasal infection with 3×10^4^ PFU MA15 virus in 25 µl DMEM. Weight loss and mortality were monitored daily. *n* = 12 mice in PBS group; 20 mice in CL group. (B) For virus titers, lungs were homogenized and titeted on Vero E6 cells. Viral titers are expressed as PFU/g tissue. *n* = 4 mice/group/time point. *P values of <0.05. (C) BALB/c mice were treated with CL or PBS 18–24 h prior to infection with 3×10^4^ PFU MA15 virus. Lungs were removed at the indicated time points p.i.. Lungs were fixed in zinc formalin, and paraffin embedded. Sections were stained with hematoxylin and eosin.

To determine whether there was a change in clinical disease after AM depletion, BALB/c mice were treated with 75 µl of CL and infected i.n. with 3×10^4^ PFU of MA15 virus. Mice were monitored daily for weight loss and mortality. At this virus dosage, control mice lost more than 20% of their body weight and 60%–70% of them died (Fig. 1 A), generally from day 6 to day 8 post infection (p.i.). Depletion of AM before inoculation (at day −1 and day −2) completely protected mice from this lethal infection and animals rapidly regained their body weight (Fig. 1 A). AM depletion at day 2 p.i. was not protective and may have resulted in more severe disease, as observed also in influenza A virus-infected mice [16]. Of note, 6 week old C57Bl/6 mice are resistant to MA15 infection and treatment at day −1 or 2 with clodronate had no effect on the clinical course in these mice (data not shown).

**Figure 2:**
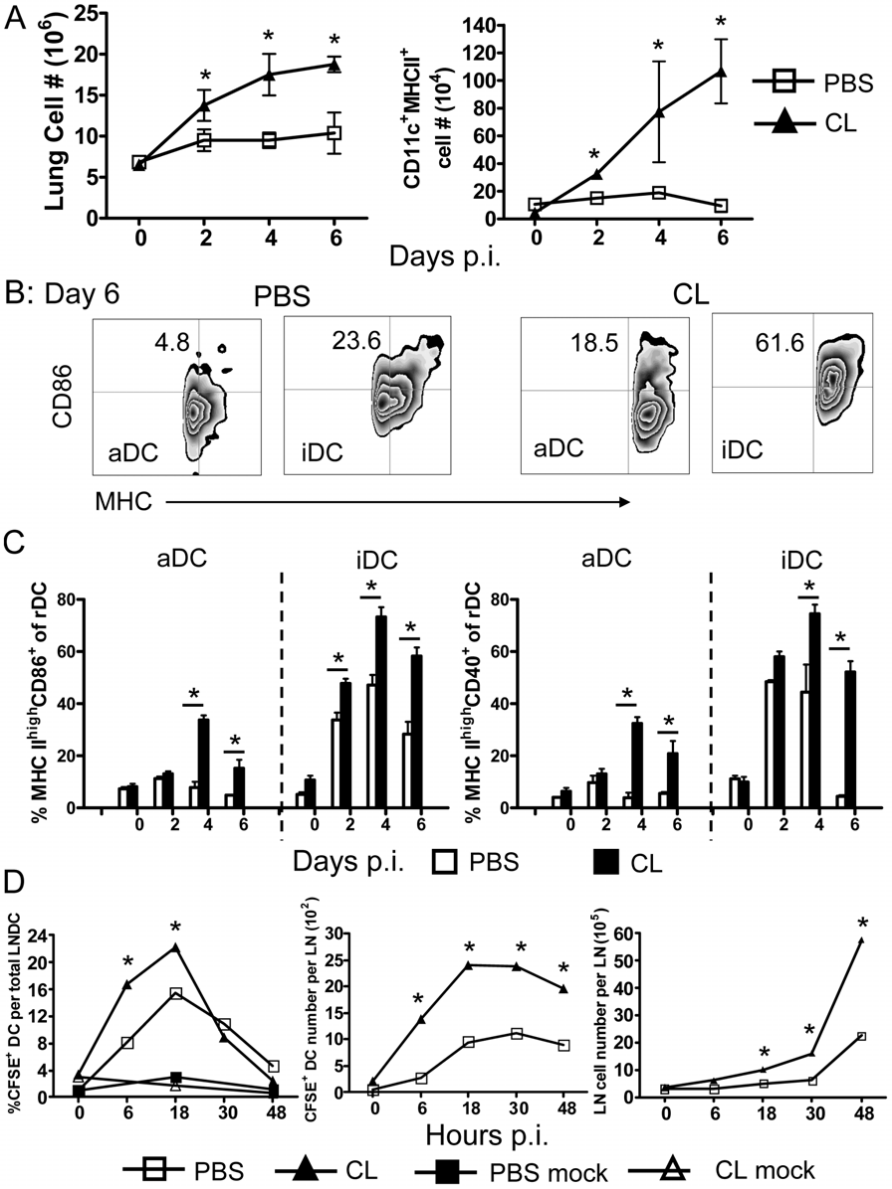
Respiratory dendritic cell recruitment, migration and activation in MA15-infected mice after CL or PBS treatment. Mice were treated with CL or PBS 18–24 h prior to infection with 3×10^4^ PFU MA15. Lungs were harvested at the indicated time points, and after enzyme digestion, single cell suspensions were acquired. Cells were stained for CD11c, MHC class II, CD11b, CD86 and CD40 expression. Total numbers of inflammatory cells and of CD11c^+^MHC II^+^ rDC in the lung are shown (A). CD86 and CD40 expression was measured on aDCs (CD11c^+^CD11b^−^MHC II^+^) and iDCs (CD11c^+^CD11b^+^MHC II^+^). An example of CD86 expression at day 6 p.i. (B) and a summary of MHC^high^CD86^+^ or MHC^high^CD40^+^ expression frequencies (C) are shown. Data are representative of two independent experiments and are the mean values±SEM (*n* = 7–8 mice/group/time point). (D) Mice were treated with CL or PBS 18–24 h before i.n. inoculation of 50 µl 8 mM CFSE. 6 h after CFSE instillation, mice were infected with 3×10^4^ PFU MA15 virus or were mock infected. At the indicated time points p.i., single cell suspensions were prepared from lung DLNs and gated for CD11c expression by flow cytometry. The values represent the percentage of CFSE^+^ cells within the CD11c^+^ DC population per LN. *n* = 4 mice/group/time point. *P values of <0.05.

Clodronate treatment resulted in enhanced kinetics of virus clearance, with virus cleared from all treated but not control BALB/c mice by day 7 p.i. (Fig. 1 B). We next examined lung sections for changes in histology. There were no histological differences in the lungs between CL-treated and control mice at day 0, indicating that depletion of AMs did not result in significant inflammatory cell recruitment to the lung. From day 2 p.i., PBS-treated mice developed a rapidly progressive interstitial pneumonia with extensive edema and damage to bronchiolar and alveolar epithelial cells (Fig. 1 C). Inflammatory infiltrates were consistently identified from days 2-to 6 p.i. CL-treated mice had a much better outcome with less destruction of the pulmonary architecture, but extensive alveolar, interstitial and perivascular inflammatory cell infiltration (Fig. 1 C, day 4 and day 6). Total lung cell numbers are shown in Fig. 2 A. Clodronate treatment, by removing AM, also altered the inflammatory milieu of the lungs. As a consequence, levels of pro-inflammatory cytokines and chemokines, such as IL-1β, Il-6, IL-12, CCL2 and CCL3 increased within 24 hours of CL, but not PBS treatment, prior to virus infection. By day 2 p.i., levels of these cytokines were generally similar in CL and PBS-treated mice, consistent with the notion that a delayed, and possibly dysregulated, immune response contributed to severe disease in control mice (Table S1).

### AM depletion enhanced rDC activation, migration and recruitment

Infection with respiratory viruses such as influenza A virus and respiratory syncytial virus (RSV) results in recruitment of CD11c^+^MHC II^+^ DCs to the lung [12],[35],[36],[37]. Unlike these infections, recruitment of inflammatory cells, including DCs, to the lung is impaired in MA15-infected mice (Fig. 2 A). The total lung cell number increased slightly, but there was no appreciable change in numbers of the respiratory dendritic cells (rDC) in control mice. Clodronate treatment resulted in enhancement of inflammatory cell recruitment to the lung (Fig. 1 C and 2 A), with a nearly tenfold increase in numbers of rDCs within 6 days (Fig. 2 A). For these experiments, we distinguished two populations of rDCs: alveolar/airway dendritic cells (aDC: CD11c^+^CC11b^−^MHC II^+^) and interstitial dendritic cells (iDC: CD11c^+^CD11b^+^MHC II^+^) using the gating strategy shown in Fig. S1. By day 4 p.i., the frequencies of MHC II^high^/CD86^+^ and MHC II^high^/CD40^+^ aDC and iDC increased significantly in drug-treated mice but only modestly on iDC and not at all on aDC in PBS-treated mice Over the next few days aDCs and iDCs remain activated in CL-treated mice but mostly returned to a baseline state in control mice (Fig. 2 B and C). Concomitant with this recruitment and activation of rDCs, we also observed enhanced rDC migration to draining lymph nodes (DLN), using a tracking method in which rDCs are labeled in the lung by i.n. inoculation of carboxyfluorescein diacetate succinimidyl ester (CFSE) (see Materials and Methods and Fig. S1 B for gating) [12]. In all mice, rDC migration to the DLNs peaked at 18 h p.i., but migration was accelerated by treatment with clodronate. After 48 hours the frequency and number of CFSE^+^ rDCs in the DLNs decreased suggesting that the first 48 h p.i. were most important period for rDC migration. There was also a two-three fold increase in total cell numbers in the DLNs (Fig. 2 D). Collectively, these results show that DCs remained activated for longer times in the lung and exhibited enhanced migration to DLNs after CL treatment. A consequence of the increase in both numbers of rDCs and the frequency that was activated was a 30–50 fold increase in total activated DCs in the lung.

### AM depletion before infection results in enhanced T cell responses

Since enhanced rDC migration to DLNs is predicted to result in enhanced virus-specific T cell responses, we next examined the magnitude of total and MA15-specific T cell responses in the lungs of CL treated and control infected mice. Clodronate treatment resulted in greater numbers of activated CD8 and CD4 T cells in the MA15-infected lung (Fig. S4 A and B), compared to PBS treatment, as determined by CD43 (clone 1B11) expression. The latter is upregulated on activated effector T cells [38],[39].

To assess effects on MA15-specific T cell responses, we initially identified a set of H-2^d^-restricted virus-specific CD4 and CD8 T cells epitopes using lung derived cells harvested from infected mice and a peptide library covering all four structural proteins (S, N, M, E) of SARS-CoV. Several IFN-γ inducing CD8 and CD4 epitopes in the spike (S) and nucleocapsid (N) proteins (S366–374, S521–529, S1061–1071 and N353–370) were identified (manuscript in preparation). Some of these epitopes were described previously, but S521 and S1061 epitopes were newly discovered. Of note, all other previously described H-2^d^-restricted T cell epitopes were not recognized by lung-derived T cells in our assays [40],[41],[42]. These previous reports identified T cell epitopes using adenovirus vectors or DNA constructs expressing single SARS-CoV proteins, or isolated peptides. We speculate that the numbers of T cells recognizing these previously described epitopes are present at very low levels in infected mice compared to the immunodominant epitopes that we identify, possibly because of differences in antigen presentation between infected and immunized mice.

**Figure 3:**
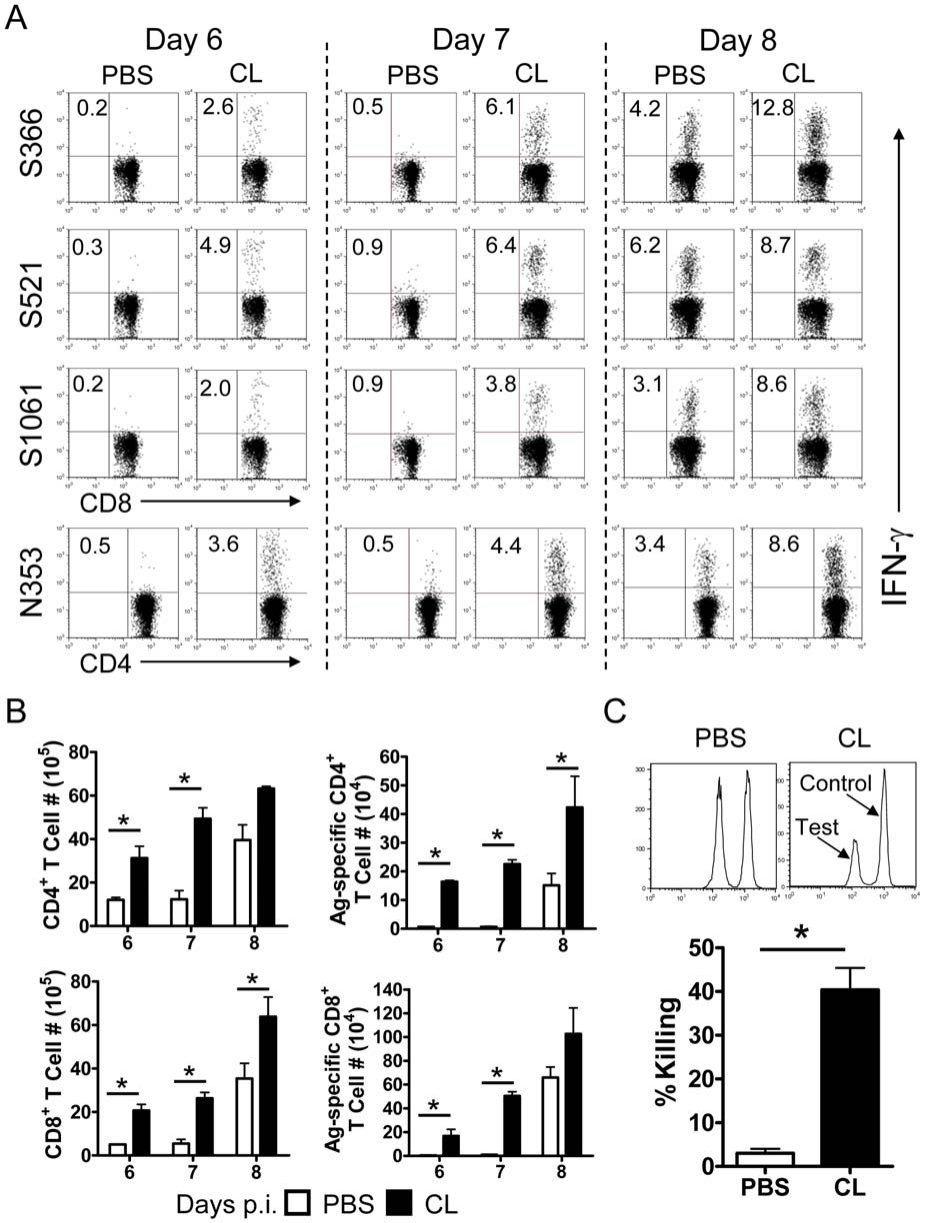
MA15-specific T cell responses in the lungs after CL treatment. Mice were treated with CL or PBS, 18–24 h prior to infection with 3×10^4^ PFU MA15 virus. At the indicated time points, single cell suspension were prepared from lungs, and stimulated with SARS-CoV CD8 (S366, S521 and S1061) or CD4 (N353) T cell peptides for 6 h in the presence of brefeldin A. Frequencies (A) and numbers (B) of total and MA15-specific T cells (determined by IFN-γ intracellular staining) are shown. Data are representative of two to four independent experiments *n* = 5–8 mice/group/time point. (C) *In vivo* cytotoxicity assays were performed on day 6 p.i.. Target cells were co-stained with PKH26 and different concentrations of CFSE (0.1 µM or 1 µM) and then incubated with SARS-CoV specific CD8 T cell peptides (0.1 µM CFSE) or in the absence of added peptides (1 µM CFSE) at 37°C for 1 h. 5×10^5^ target cells from each group were mixed together (1×10^6^ in total) and transferred i.n. to infected mice. 12 h after transfer, single cell suspensions were prepared from the lung and examined by flow cytometry. *n* = 3–4 mice/group. Data are representative of two independent experiments. *P values of <0.05.

Using these epitopes, we found that AM-depleted mice exhibited earlier and more robust virus-specific T cell responses, as measured by intracellular cytokine staining (ICS) for IFN-γ, whereas control mice had almost no virus-specific T cell responses at days 6 and 7 p.i. (Fig. 3 A and B). PBS-treated mice that survived until day 8 p.i. mounted virus-specific T cell responses in the lung, but at a level that was much less than observed in CL-treated mice. We confirmed that these cells were functional using *in vivo* cytotoxicity assays. Naïve splenocytes were costained with PKH26 and CFSE, pulsed with MA15-specific CD8 T cell peptides and adoptively transferred i.n. into mice 12 h before harvest of total lung cells. Robust CD8 T cell cytotoxic responses were observed in AM-depleted mice, with 40%–50% killing of virus-specific targets. By comparison, only about 5% of target cells were lysed in control mice (Fig. 3 C).

### Alveolar macrophages are inhibitory in vivo and *in vitro*

**Figure 4:**
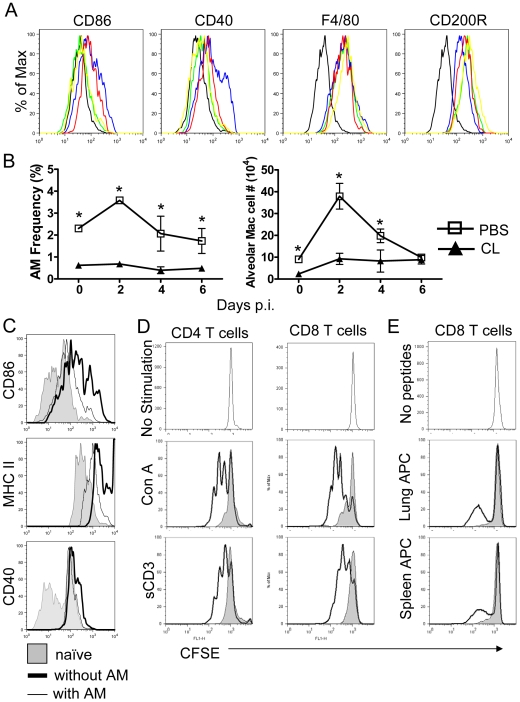
Phenotype and numbers of AM in MA15-infected lungs after treatment with CL or PBS and AM-mediated inhibition of aDC activation and T cell proliferation *in vitro*. Mice were treated with CL or PBS at day −1 prior to infection with 3×10^4^ PFU MA15. CD86, CD40, F4/80 and CD200R expression on CD11c^+^CD11b^−^Siglec F^+^ AM (A) and numbers and frequency (B) of AM were determined by flow cytometry. Black, isotype control; green, naive; blue day 2; red, day 4. yellow, D6. Data are representative of two independent experiments and are the mean values±SEM (*n* = 7–8 mice/group/time point). *P values of <0.05. (C) To assess the ability of AM to suppress aDC activation *in vitro*. AMs were harvested from BAL (bronchoalveolar) fluid and cultured at 4×10^4^/well in 96-well dishes for 48 h before use. aDCs were purified from naïve mice lungs by FACS sorting. aDCs were cultured in the presence or absence of AMs together at a 1∶1 ratio for 24 h at 37°C and subjected to flow cytometry. Data are representative of four independent experiments. (D) To assess the ability of AM to inhibit T cell proliferation *in vitro*, single cell suspensions were prepared from the lungs of naïve mice or CL-treated MA15-infected mice at day 8 p.i. Cells were incubated on plastic dishes for 2 h at 37°C, to remove AMs. Naïve lung cells (4×10^5^/96-well) were stained with 1 µM CFSE and stimulated with either 2.5 µg/ml Con A or 1 µg/ml soluble CD3 antibody with or without AMs (4×10^4^ / 96-well) for 72 h. Solid line, without AMs; gray, with AMs. (E) Total CD8 T cells were purified by microbeads from lung cells of AM-depleted MA15-infected mice at day 8, stained with 1 µM CFSE and stimulated with splenocytes from naïve mice or CD8 T cell-depleted infected lung cells (4×10^5^/96-well) that were pulsed with SARS-CoV CD8 T cell peptides with or without AM (4×10^4^ / 96-well) for 72 h. Cells were then subjected to flow cytometry. Solid line, without AMs; gray, with AMs. Data are repesentative of two independent experiments.

Results thus far suggest that inhibitory macrophages are dominant in MA15-infected lungs. In support of this, AM were only transiently and slightly activated, as measured by CD86 and CD40 expression, after infection with MA15 (Fig. 4 A). F4/80, considered a marker for macrophage maturation and phagocytosis [43], was present at lower levels on AMs harvested from uninfected mice compared to macrophages isolated from other sites (e.g., peritoneal macrophages [44], Fig. S5) and was not upregulated after MA15 infection (Fig. 4 A). Further, surface levels of CD200R, important in maintaining lung homeostasis, were higher on AM than peritoneal macrophages [44] (Fig. S5) and were not significantly downregulated after infection (Fig. 4 A), indicating that AMs continued to be inhibitory even after the onset of the infection. The number and frequency of AMs increased at day 2 before returning to baseline by day 6 p.i. in control mice but, as expected, remained low throughout the infection after clodronate treatment, (Fig. 4 B).

Mature “resting” AMs are able to suppress *in vitro* proliferation of homologous T-cells, and freshly isolated rDCs are poor antigen-presenting cells, consistent with a suppressive state [6],[45]. To confirm the inhibitory properties of AMs, we isolated aDCs from total lung cells and cultured them *in vitro* for 24 h in the presence and absence of AMs. When cultured in the absence of AMs, aDC upregulated expression of CD86, MHC II and CD40. Co-culture with AMs prevented CD86 and MHC class II, and to a lesser extent, CD40 upregulation (Fig. 4 C).

The prolonged presence of AMs in MA15-infected lungs suggested that AMs not only inhibited rDCs activation, and thereby delayed DC migration from lung to lymph nodes, but also inhibited the function of anti-virus T cells in the lung. To examine this possibility, we co-cultured AMs and T cells *in vitro*. Concanavalin A (Con A) and soluble anti-CD3 (sCD3) antibody treatment of lung cells resulted in proliferation of both CD4 and CD8 T cells as measured by CFSE dilution. This proliferation was almost completely inhibited by co-culture with purified AMs at a ratio of 10∶1 (10 T cells∶1 AM) (Fig. 4 D). Of note, endogeous AMs were removed from the lung cell preparations by incubation in a tissue culture plate for 2 h (90% depletion, measured by flow cytometry). In the absence of this prior incubation, no robust proliferation was observed. To assess the effect of AM on virus-specific T cells, we isolated CD8 T cells from MA15-infected, CL-treated mouse lungs at day 8 p.i. using microbeads and stained them with CFSE. Cells were then stimulated for 72 hours with lung cells or splenocytes that were pulsed with three MA15-specific CD8 T cell peptides (S366/S521/S1061) with or without AMs. Although only about 30% of CD8 T cells were MA15-specific, proliferation of CD8 T cells was clearly detected. When co-cultured with AMs, CD8 T cell proliferation was totally inhibited (Fig. 4 E). Thus, AMs inhibited both nonspecific and specific CD8 T cell proliferation. However, AM co-culture *in vitro* did not inhibit IFN-γ expression after stimulation with MA15-specific peptides (Fig. S6 A), consistent with previous data, showing that AMs did not inhibit IL-2 secretion by Con A-stimulated T cells [45]. Further, when AMs and T cells were separated by a transwell during co-culture, no significant decrease of proliferation was observed as measured by CFSE dilution (Fig. S6 B) suggesting that AM inhibition of T cell proliferation required direct cell contact.

### Poly I:C treatment protected mice from lethal MA15 infection

**Figure 5:**
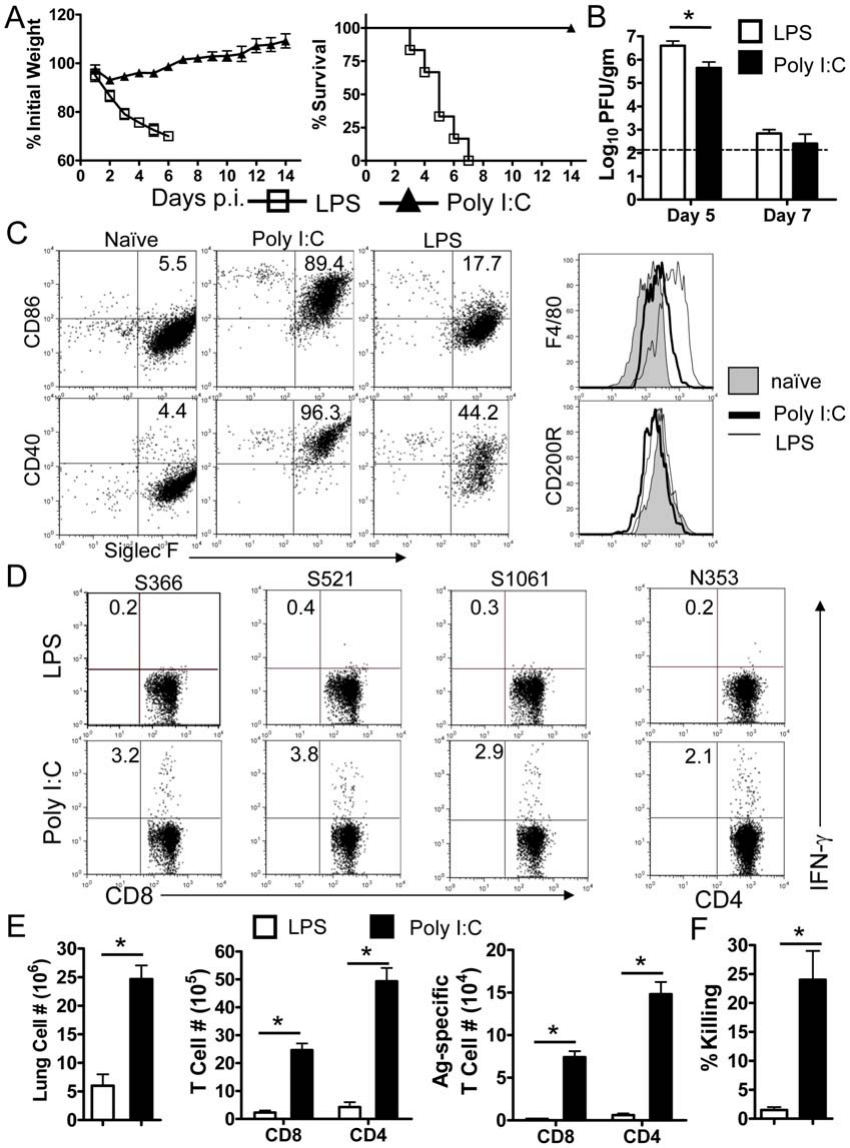
Protective effects of poly I:C treatment. (A) Mice were treated with 20 µg poly I:C or 5 µg LPS 18–24 h before infection with MA15. Weight loss and mortality were monitored daily. *n* = 18 in LPS group; 14 mice in Poly I:C group. (B) Lungs were harvested and homogenized and virus was titered on Vero E6 cells. Viral titers are expressed as PFU/g tissue. (*n* = 4 mice/group/time point). (C) Single cell suspensions were prepared from lungs of naïve and treated mice. CD86, CD40. F4/80 and CD200R expression by CD11c^+^CD11b^−^Siglec F^+^ AMs after poly I:C, LPS or no treatment was determined by flow cytometry. The frequencies of MHC II^+^ CD86^+^ cell populations are shown. (D and E) Mice were treated with poly I:C or LPS 18–24 h prior to MA15 infection. At day 7 p.i., single cell suspensions were prepared from lungs, and stimulated with SARS-CoV CD8 (S366, S521 and S1061) or CD4 (N353) T cell peptides for 6 h in the presence of brefeldin A. Cells were analyzed for IFN-γ expression. Frequency (D) and numbers (E) of virus specific T cells are shown. Data are representative of two independent experiments and are the mean values±SEM (*n* = 5–8 mice/group/time point). (F) *In vivo* cytotoxicity assays were performed on day 6 p.i. Target cells were co-stained with PKH26 and different concentrations of CSFE, then pulsed with/without SARS-CoV specific CD8 T cell peptides, mixed together (1×10^6^ in total) and transferred i.n. to mice. 12 h after transfer, lung cells were examined by flow cytometry. *n* = 3–4 mice/group. Data are representative of two independent experiments.

The results described above raised the possibility that direct activation of rDCs in the lung or adoptive transfer of activated DCs to the lung would bypass AM inhibitory function. Signaling through Toll-like receptors (TLR) results in a series of signaling events that leads to the induction of an acute inflammatory response. Ligand binding to TLRs also results in dendritic cell maturation, which is necessary for the initiation of adaptive immune responses [46],[47],[48]. Previous reports showed that Poly I:C or CpG treatment protected animal from lethal virus infection, but the mechanism of protection was not investigated in those studies [49],[50]. In preliminary experiments, we treated mice with ligands for several TLRs, including poly I:C (TLR3), LPS (TLR4), CpG (TLR9), R837(TLR7), R848 (TLR7/8), Pam_3_CSK4 (TLR1/2), and Pam_2_CSK4 (TLR2/6). We observed that treatment with poly I:C (Fig. 5 A) and, to a lesser extent, CpG (data not shown), but not the other TLR ligands, protected mice from lethal disease. Consequently, additional analyses were performed after treatment with poly I:C and as a control, LPS since both are widely used to stimulate macrophages and DCs [51]. Poly I:C (20 µg/mouse)-treated mice lost about 10% of their original weight but quickly recovered within 7 days. The LPS-treated group (5 µg/mouse), lost more than 20% of their weight with death occurring in all mice within 6–7 days. Virus titers were higher at day 5 in the lungs of these mice compared to mice treated with poly I:C (Fig. 5 B).

**Figure 6:**
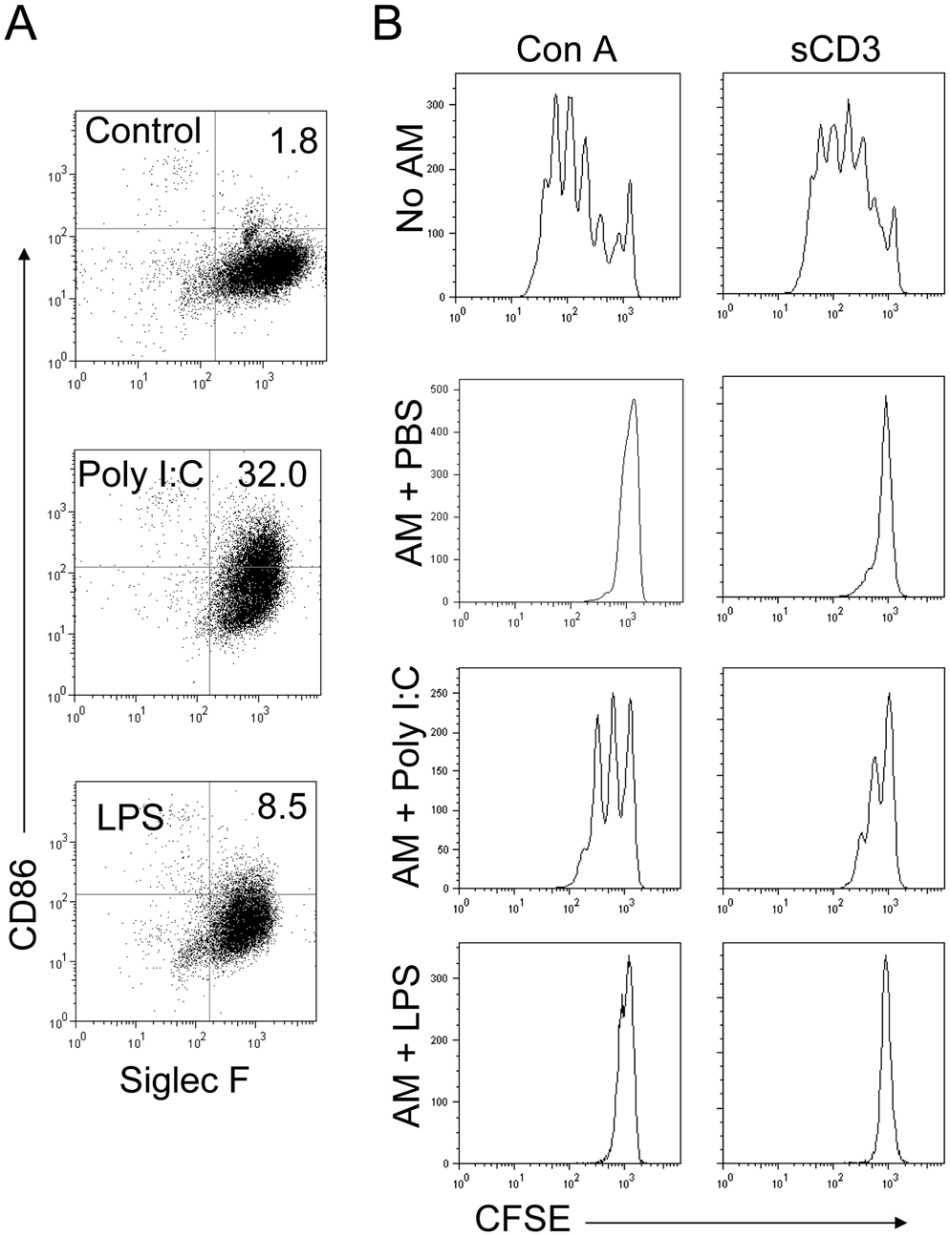
Poly I:C treatment partially reverses AM inhibition of T cell proliferation *in vitro*. (A) AMs were harvested from BAL fluid, and cultured at 2.5×10^5^/well in 24-well dishes for 48 h in the presence of 20 µg/ml poly I:C or 1 µg/ml LPS. Cells were detached and subjected to flow cytometry. (B) Single cell suspension were prepared from spleens of naïve mice, stained with 1 µM CFSE and stimulated with either 2.5 µg/ml Con A or 1 µg/ml soluble CD3 antibody for 72 h in the presence or absence of poly I:C-stimulated AMs from (A). Samples were then subjected to flow cytometry. Data are representative of two independent experiments.

Poly I:C, and to a much less extent LPS treatment resulted in enhanced CD86 and CD40 upregulation on AMs (Fig. 5 C) and rDCs (Fig. S7). Treatment with both TLR agonists resulted in a modest increase in F4/80 and a small decrease in CD200R expression (Fig. 5 C). Consistent with the results obtained after clodronate treatment (Fig. 3 A), poly I:C treatment resulted in an earlier and more robust antigen-specific T cell responses than observed in PBS (Fig. 3 A) or LPS-treated mice (Fig. 5 D, E). Nearly twenty fold more MA15-specific T cells were detected in the lungs of poly I:C treated mice compared to LPS recipients at day 7 p.i. and these cells were functional in *in vivo* killing assays (Fig. 5 E and F). To determine whether poly I:C or LPS directly activated AMs, AMs were isolated and stimulated *in vitro* with both agonists. After 24 h stimulation, poly I:C but not LPS treatment resulted in a pronounced upregulation of CD86 (Fig. 6 A). Further, treatment with poly I:C but not LPS partially reversed the ability of AM to inhibit CD8 T cell proliferation after stimulation with Con A or sCD3 (Fig. 6 B). These results indicate that poly I:C can abrogate AM inhibitory function both *in vivo* and *in vitro*, by directly activating AMs and rDCs.

### Adoptive transfer of activated BMDCs protected mice from lethal MA15 infection

**Figure 7:**
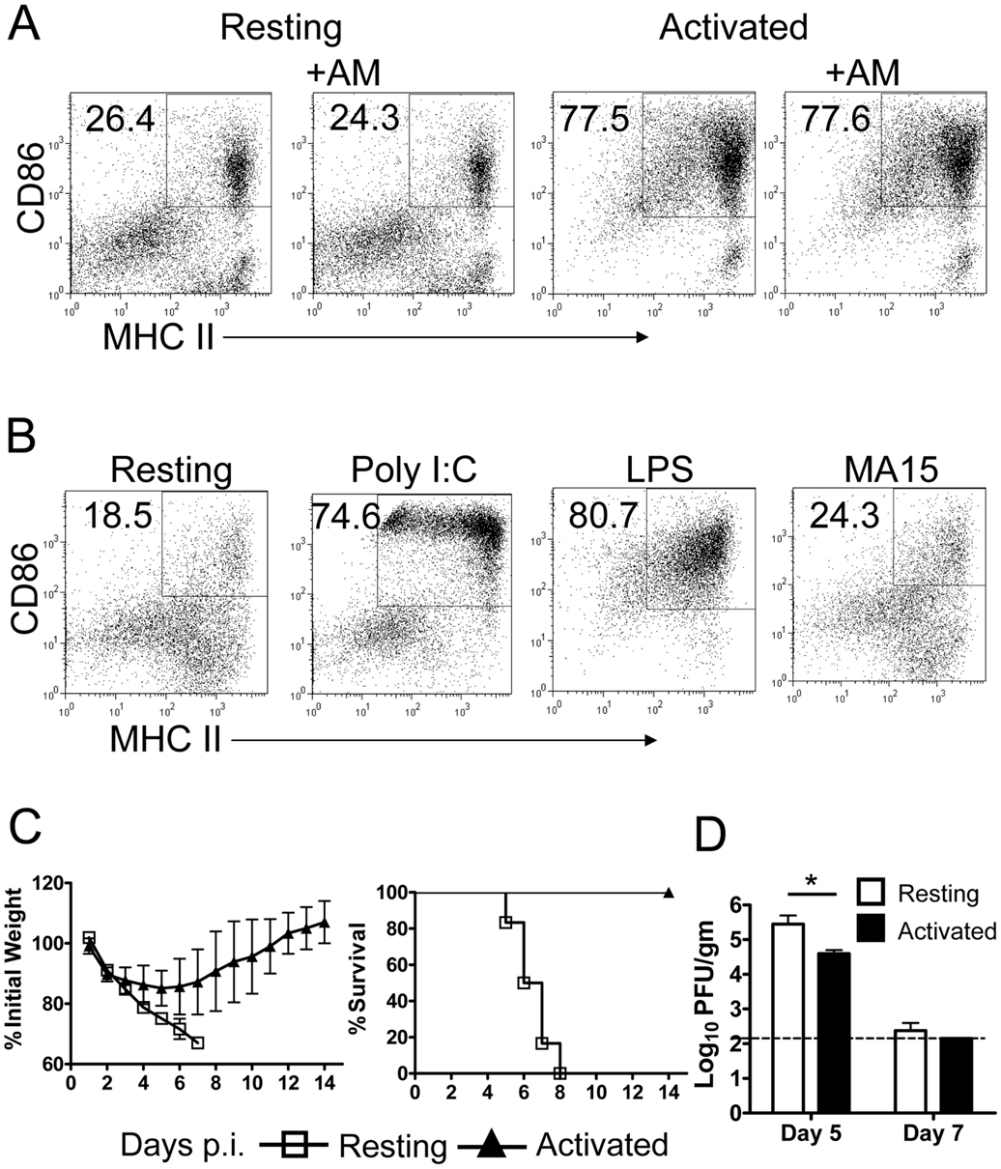
Activation of BMDCs and protective effect of adoptive transfer of activated but not resting BMDCs. (A) LPS (1 µg/ml) activated BMDCs were co-cultured with AMs harvested from BAL of naïve mice for 24 h. Phenotype changes were assessed by flow cytometry. AM co-culture did not inhibit costimulatory molecule expression by previously activated BMDCs. (B) BMDCs were stimulated with 20 µg/ml poly I:C or 1 µg/ml LPS or MA15 virus (m.o.i. = 5) and assayed for CD86 expression. Both poly I:C and LPS activated AM, as measured by CD86 expression. (C) 3×10^5^ activated or resting BMDCs were transferred by i.n. inoculation 18 h before MA15 infection (3×10^4^ PFU/mouse). Weight loss and mortality were monitored daily. *n* = 12 mice in resting BMDC group; 15 mice in activated BMDC group. (D) Lungs were homogenized and virus titered on Vero E6 cells. Viral titers are expressed as PFU/g tissue. (*n* = 4 mice/group).

**Figure 8:**
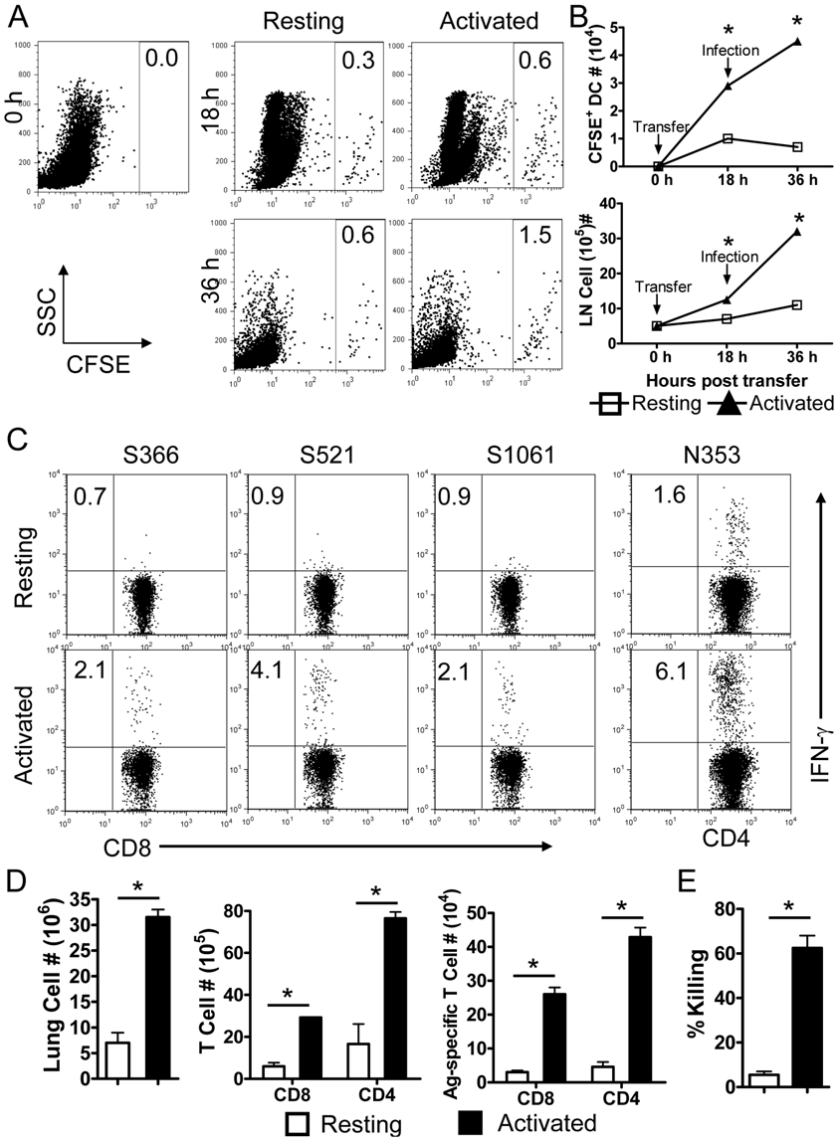
Enhanced DC migration to DLN and MA15-specific T cell response after. transfer of activated but not resting BMDCs. (A) Activated or resting BMDCs were stained with 1 µM CFSE, and adoptively transferred to mice. After 18 h, mice were infected with 3×10^4^ PFU MA15. Single cell suspensions were prepared from DLNs and CFSE^+^ cells were identified by flow cytometry. Total CFSE^+^ cells and LN cells numbers are shown in (B). Activation of BMDC enhanced migration to DLN and also increased total DLN cellularity. Data are representative of two independent experiments and are the mean values±SEM (*n* = 6–8 mice/group/time point). (C and D) Activated or resting BMDCs were transferred 18–24 h prior to infection with MA15. At day 7 p.i., single cell suspensions were prepared from lungs, and stimulated with SARS-CoV CD8 (S366, S521 and S1061) or CD4 (N353) T cell peptides for 6 h in the presence of brefeldin. Cells were analyzed for IFN-γ expression A. Frequency (C) and numbers (D) of MA15-specific T cells are shown. Data are representative of two independent experiments and are the mean values±SEM (*n* = 6–7 mice/group/time point). (E) *In vivo* cytotoxicity assays were performed on day 6 p.i. Target cells were co-stained with PKH26 and different concentrations of CSFE, pulsed with/without SARS-CoV specific CD8 T cell peptides, mixed together (1×10^6^ in total) and transferred i.n. to mice. 12 h after transfer, lung cells were examined by flow cytometry. *n* = 3–4 mice/group. Data are representative of two independent experiments.

Given these results, direct delivery of activated DCs to the lungs might overcome AM-mediated inhibition. Activated DCs exhibit an enhanced ability to migrate to DLNs and to stimulate CD8 T cell proliferation and IFN-γ expression [52],[53],[54],[55]. Since AMs were unable to inhibit costimulatory molecule expression on previously activated DCs (Fig. 7 A), we next assessed whether adoptively transferred activated DCs could bypass AM inhibition and protect mice from a lethal MA15 infection. For this purpose, bone marrow cells were harvested from naïve mice, and DCs selectively cultured by treatment with GM-CSF plus IL-4 for 6 days [56]. BMDCs were then activated with either LPS or poly I:C, which resulted in enhanced CD86 and MHC class II expression on BMDCs (Fig. 7 B). As expected, MA15 was unable to activate these cells. 3×10^5^ activated or resting BMDCs were transferred to mice i.n. 18 h prior to infection. Mice that received BMDCs activated with either poly I:C or LPS were protected from a fatal outcome, although they still lost about 15% of their body weight. In marked contrast, recipients of resting BMDC were not protected (Fig. 7 C). Further, higher virus titers were detected in the lungs at day 5 mice that received resting BMDC as opposed to activated BMDC (Fig. 7 D). BMDC migration from the lungs to DLNs was accelerated by prior activation. More CFSE^+^ activated BMDC than resting BMDC accumulated in the DLNs of recipient mice (Fig. 8 A and B) and additionally, the total number of cells in the DLNs was increased dramatically by activated BMDC transfer (Fig. 8 B). Consistent with enhanced rDC migration to the DLNs, recipients of activated BMDCs developed more robust CD4 and CD8 T cell responses in the lungs when compared to those that received resting BMDC (Fig. 8 C and D). Nearly tenfold more MA15-specific T cells were detected in the lungs of activated BMDC compared to resting BMDC recipients at day 7 p.i. and these cells were functional in *in vivo* killing assays (Fig. 8 E). Collectively, these results indicate that adoptive transfer of activated BMDCs to the lung amplified virus specific T cell responses, cleared virus earlier and protected mice from lethal MA15 infection.

## Discussion

The pathogenesis of SARS in patients that exhibit more severe disease is not well understood but includes slow virus clearance and a prolonged clinical course [19],[21],[57],[58]. The results presented herein suggest that this severe disease may occur in part because infected individuals do not mount an appropriate anti-virus T cell response. Anti-virus CD8 T cells are critical for virus clearance in mice infected with other pathogens, such as influenza A virus and LCMV [15],[59], so it is not unexpected that they are necessary for resolution of infection with SARS-CoV. While lymphopenia is associated with a worse prognosis in SARS patients [17],[18],[19], no prior studies, to our knowledge, has shown that this poor prognosis results, in part, from a sub-optimal CD8 T cell response. This defect in development of a protective T cell response occurs because the virus does not reverse the anti-inflammatory state that is naturally present in the uninfected lung. These results are consistent with *in vitro* studies in which the SARS-CoV is able to infect but can not activate human DCs or macrophages [22],[23],[24],[25],[26],[27],[28]. This may occur, in part, because coronaviruses, including SARS-CoV, are “invisible” to cellular sensors in some cell types [29].

Alveolar macrophages play a central role in maintaining immunological homeostasis [1],[2] and actively suppress the induction of adaptive immunity through their effects on alveolar and interstitial DCs and T cells [6],[7],[8]. Several molecules, including nitric oxide, TGF-β and CD200R have been implicated in AM suppressive function. These molecules have either short half lives or require cell-to-cell contact [1],[44],[60],[61]. Consistent with this, AMs are separated by a distance of only 0.2–0.5 µm from rDCs in the lung [10]. Our results also suggest that cell contact or close proximity to target cells is required, because AMs were unable to suppress T cell proliferation if separated from responders by a transwell membrane (Fig. S6 B). In another mechanism that maintains an anti-inflammatory state in the lungs, AMs ingest and process innocuous antigen and bacteria before they can reach and activate rDCs [10]. AM depletion results in enhanced antigen-presenting function by rDCs [6] and in increased ability to lyse influenza A virus-infected cells [62].

These reports indicate that countering the quiescent, anti-inflammatory state of AM is critical for developing a protective immune response; our results indicate that infection with SARS-CoV reverses this quiescent state inefficiently. We used three approaches to support this conclusion. First, pre-treatment of MA15-infected mice with clodronate depleted AM, resulting in enhanced activation and migration of rDCs, which in turn led to the development of a vigorous and protective virus-specific T cell response in the lung (Fig. 2 and 3). The activation and migration of rDCs at early times p.i. are critical for the timely initiation of anti-SARS-CoV T cell responses. Consistent with this, treatment with clodronate at day 2 p.i. was not protective (Fig. 1), because rDC migration to the DLNs is largely complete by 48 hours p.i. ((Fig. 2 D) and [12]). Depletion at day 2 p.i. resulted in more severe disease, suggesting that in SARS-CoV-infected mice, virus-specific T cells require additional DC stimulation in the lungs, as occurs in influenza A-infected animals [16].

Second, activation of AMs and rDCs *in situ* via treatment with TLR agonists also circumvented the anti-inflammatory state of the lung. Our results showed that only poly I:C, a TLR3 agonist, and to a lesser extent CpG, a TLR9 agonist, were able to perform this function. TLR7 is primarily located on plasmacytoid DCs and the inability of R848 to protect mice indicates that activation of these cells was insufficient to induce a protective immune response. Poly I:C, which activated AMs and rDC *in vivo* (Fig. 5 C and S7) and *in vitro* (Fig. 6 A), protected animals from lethal MA15 infection. The ability of poly I:C to stimulate rDC activation and migration has been described previously [12], and is likely to explain its protective ability. It should be noted that poly I:C treatment also induced type 1 IFN expression in the lung. This may also have contributed to the protective effect of poly I:C, but this is not likely to be the major effect because SARS-CoV is only modestly sensitive to IFN treatment of cultured cells or of mice [63],[64]. In addition, CL treatment did not induce type 1 IFN in the lungs, showing that IFN induction is not required for protection (data not shown).

LPS, which is a TLR4 agonist, was unable to protect mice from lethal disease. We considered the possibility that LPS might have toxic effects unrelated to TLR4 binding, but treatment with monophosphoryl lipid A (MPLA), a derivative of LPS that is a TLR4 agonist but is less toxic [65],[66], was also not protective (data not shown). Our results are consistent with a recent study that showed that TLR4 ligation contributed to worse outcomes in several models of lung injury [67]. TLR4 ligation, in the absence of treatment with specific agonists, did not contribute to worsened disease in MA15-infected BALB/c mice since infection of TLR4^−/−^ BALB/c mice did not result in significant differences in clinical disease when compared to wild type BALB/c mice (data not shown).

Third, we showed that adoptive transfer of activated but not resting BMDCs bypassed AM-mediated suppression and protected mice from lethal disease (Fig. 7). While DC maturation makes these cells the most potent in antigen presentation in an animal, it also results in the loss of ability to take up antigen. However, antigen macropinocytosis is transiently stimulated after activation [52], possibly explaining how transferred BMDC could acquire SARS-CoV antigen for presentation to T cells in the DLNs. Alternatively, mature DCs are able to uptake antigen for cross-presentation [68]. Activated BMDCs preferentially migrated to the DLNs (Fig. 8 A and B) and initiated a protective T cell response in the lungs (Fig. 8 C–E). This transfer was successful because inhibitory AMs cannot reverse prior rDC activation (Fig. 7 A). All of these three experimental interventions resulted in enhanced rDC migration to the DLNs, enhanced MA15-specific T cell responses at the site of infection, the lungs, and improved outcomes.

It is notable that virus-specific T cells are also critical for virus clearance in C57BL/6 mice, which are resistant to MA15 infection. Six week old mice deficient in recombination activating enzyme activity 1 (RAG1^−/−^) on a C57Bl/6 background do not clear virus when measured at 9 days [69] or even 21 days p.i. (data not shown), yet remain completely asymptomatic. On the other hand, mice with Severe Combined Immunodeficiency Syndrome (SCID) on a BALB/c background, which, like RAG1^−/−^ mice, are genetically unable to mount a T cell response, develop clinical disease that is more severe than that observed in wild type BALB/c mice. All SCID mice succumb to the infection (data not shown), compared to a 60–70% mortality rate in BALB/c mice that are infected with the same dosage of virus (Fig. 1 A). Collectively, these results show that an optimal T cell response is required for virus clearance but that strain-specific components of the initial immune response, not yet defined, are critical for preventing clinical disease in resistant strains.

An outstanding question is why SARS-CoV does not activate AMs and rDCs in BALB/c mice. As described above, SARS-CoV does not efficiently activate human DCs or macrophages. We have also shown that MA15 does not efficiently induce costimulatory molecule upregulation on murine rDCs or AM *in vivo* and/or *in vitro* (Fig. 2 C, 4 A and S2). However, while most viruses have mechanisms to evade host recognition sensors, they still efficiently induce an immune response. For example, successful resolution of influenza A virus infections requires activation of immune responses via TLR7, RIG-I and NLR (NOD-like receptors) inflammasome pathways [70], even though influenza A virus encodes an immune-evading protein, nsp1 [71]. HSV, lymphocytic choriomeningitis virus, hepatitis C virus, RSV and human cytomegalovirus are recognized via TLR2-dependent mechanisms while the RSV F protein activates cells via a TLR4-dependent mechanism [37]. Some viruses, such as vaccinia virus, directly inhibit TLR expression, confirming the importance of these molecules in virus recognition by the host [72]. TLR signaling is also important for SARS-CoV recognition by the innate immune system, since C57BL/6 mice, which are very resistant to the virus, become susceptible when MyD88 is genetically deleted [69]. The precise TLR or other receptor required for protection in C57BL/6 mice is not known at present. Why this same pathway is not efficiently induced in BALB/c mice after MA15 infection will be an area of future investigation.

In conclusion, we have shown that lethal disease in mice infected with a mouse-adapted strain of SARS-CoV (MA15) is correlated with a lack of activation of AMs and rDCs. Further, lethal disease can be prevented if AMs with anti-inflammatory properties are depleted from lungs prior to infection. Depletion results in enhanced DC recruitment to the lung and accelerated migration to DLN, and a more vigorous anti-SARS-CoV T cell response. Treatment with TLR agonists to activate AMs and rDCs or transfer of activated BMDCs also prevents a lethal outcome. Together, these results demonstrate that SARS-CoV, by “hiding” from the immune system, uses a novel mechanism to evade immune recognition in mice. The pathogenesis of SARS in humans may involve similar stealth mechanisms.

## Materials and Methods

### Mice, cells and virus

Pathogen-free BALB/c mice were purchased from the National Cancer Institute (Frederick, MD). Mice were maintained in the animal care facility at the University of Iowa. Animal studies were approved by the University of Iowa Animal Care and Use Committee. African Green monkey kidney-derived Vero E6 cells were grown in Dulbecco's modified Eagle's medium (DMEM, GIBCO, Grand Island, NY) supplemented with 25 mM HEPES and 10% fetal bovine serum (FBS) (Atlas Biologicals, Fort Collins, CO). Mouse-adapted SARS-CoV (MA15) was a kind gift from Dr. Kanta Subbarao (N.I.H., Bethesda, Maryland) [30]. Virus was passaged once on Vero E6 cells.

### Virus infection and titration

Mice were lightly anesthetized with isoflurane and infected intranasally (i.n.) with 3×10^4^ PFU of MA15 virus in 25 µl of DMEM medium. Mice were monitored for weight loss and mortality daily. All work with MA15 virus was conducted in the University of Iowa Biosafety level 3 (BSL3) Laboratory Core Facility. To obtain lungs for virus titers, animals were sacrificed at the indicated time points post-infection (p.i.) and lungs were removed into phosphate buffered saline (PBS). Tissues were homogenized using a manual homogenizer, and titered on Vero E6 cells. For plaque assays, cells were fixed with 10% formaldehyde and stained with crystal violet three days post-infection. Viral titers are expressed as PFU/g tissue.

### Peptides and chemicals

A peptide library, covering all 4 structural proteins of SARS-CoV was provided by BEI Resources (Manassas, VA). Virus-specific peptides were synthesized by BioSynthesis Inc. (Lewisville, TX). TLR agonists poly I:C, Monophosphoryl Lipid A (MPLA), CpG, Imidazoquinoline compound (R837 and R848), Pam_3_CSK4 and Pam_2_CSK4 were purchased from Invivogen (San Diego, CA). LPS was purchased from Alexis Biochemicals (Farmingdale, NY).

### Clodronate-liposome treatment

Alveolar macrophage depletion was performed by treatment with liposomes containing dichloromethylene bisphosphonate (clodronate). Clodronate was a gift from Roche Diagnostics GmbH (Mannheim, Germany), and it was encapsulated in liposomes as described earlier [9],[33]. At the indicated times, mice were anesthetized by intraperitoneal injection of 2% avertin and administered 75 µl of clodronate liposomes, or PBS i.n.

### Histology

Animals were anesthetized and transcardially perfused with PBS followed by zinc formalin. Lungs were removed, fixed in zinc formalin, and paraffin embedded. Sections were stained with hematoxylin and eosin.

### Lung cells and draining lymph node cells preparation

Mice were anaesthetized with 100 µl pentobarbital (50 mg/ml, Lundbeck Inc., Deerfield, IL) at the indicated time points. The lung vascular bed was flushed via the right ventricle with 5 ml PBS to eliminate any blood and lungs and draining lymph nodes were then removed. Lungs were cut into small pieces and digested in HBSS buffer containing 2% FCS, 25 mM HEPES, 1 mg/ml Collagenase D (Roche, Indianapolis, IN) and 0.1 mg/ml DNase (Roche) for 30 min at RT. Lymph nodes were minced and pressed though a wire screen. Particulate matter was removed with a 70 µm nylon filter to obtain single-cell suspensions. Cells were enumerated by 0.2% trypan blue exclusion.

### In situ CFSE staining

CFSE (Molecular Probes, Eugene, OR) was dissolved at 25 mM in DMSO stored at −20°C until use. The CFSE stock solution was diluted in DMEM media to a concentration of 8 mM and then administered i.n. (50 µl/mouse) following anesthesia with isoflurane [12].

### Flow cytometry

The following monoclonal antibodies were used for these studies: rat anti-mouse CD3 (145-2C11), rat anti-mouse CD4 (RM4-5), rat anti-mouse CD8β (53-6.7), rat anti-mouse CD11b (M1/70), hamster anti-mouse CD11c (HL3), rat anti-mouse CD16/32 (2.4G2), rat anti-mouse Siglec F (E50-2440), mouse anti-mouse I-A^d^ (AMS-32.1), all from BD Bioscience (San Diego, CA); rat anti-mouse IFN-γ (XMG1.2), anti-mouse F4/80 (BM8), rat anti-mouse CD40 (1C10), all from eBioscience (San Diego, CA); rat anti-mouse CD43 (1B11, Biolegend, San Diego, CA); rat anti-mouse CD200R (OX-110, Serotec, Raleigh, NC).

For surface staining, 10^6^ cells were blocked with 1 µg anti-CD16/32 antibody and 1% rat serum, stained with the indicated antibodies, and then fixed using Cytofix Solution (BD Biosciences). For intracellular cytokine staining (ICS), cells were cultured at 1×10^6^ per 96-well at 37°C for 6 h or the indicated time period in the presence of brefeldin A (BD Biosciences). Cells were then labeled with surface antibodies, fixed/permeabilized with Cytofix/Cytoperm Solution (BD Biosciences) and labeled with anti-IFN-γ antibody. All flow cytometry data were acquired on a BD FACSCalibur or an LSR II (BD Biosciences) flow cytometer with CellQuest (BD Biosciences) and were analyzed using FlowJo software (Tree Star, Inc. Ash, OR).

### In vivo cytotoxicity assay

In vivo cytotoxicity assays were performed on day 6 after MA15 infection, as previously described [73]. Briefly, splenocytes from naive mice were costained with PKH26 (Sigma-Aldrich, St. Louis, MO) and either 1 µM or 100 nM CFSE (Molecular Probes, Eugene, OR). Labeled cells were then pulsed with the indicated peptides (3 µM) at 37°C for 1 h and 5×10^5^ cells from each group were mixed together (1×10^6^ cells in total). Cells were transferred i.n. into mice and at 12 h after transfer, total lung cells were isolated. Target cells were distinguished from host cells on the basis of PKH26 staining and from each other on CFSE staining. After gating on PKH26^+^ cells, the percentage killing was calculated as previously described [73].

### Alveolar macrophage preparation and *in vitro* T cell co-culture

AMs were obtained from uninfected lungs as previously described [74]. Briefly, lungs were inflated with warm PBS containing 0.2% BSA and 12 mM lignocaine (Sigma-Aldrich, St. Louis, MO) via cannulation of the trachea, and were lavaged at least 6 times. Cells were collected by centrifugation, resuspended in RPMI 1640 (Gibco, Grand Island, NY) containing 10% FCS (Atlanta, Lawrenceville, GA) and cultured at 4×10^4^ in each 96-well for 48 h before use in the presence or absence of stimulators [75].

To demonstrate inhibition of polyclonal T cell proliferation, 4×10^5^ splenocytes or lung cells (after AM-depletion by attachment to plates for 2 h at 37°C) were labeled with 1 µM CFSE and added to wells, stimulated with 2.5 µg/ml Con A (Sigma) or 1 µg/ml soluble CD3 (eBioscience) and cultured with AMs at a ratio of 10∶1 for 72 h. For inhibition of virus-specific CD8 T cell proliferation, lung CD8 T cells were purified from AM-depleted, MA15-infected animals at day 8 p.i. using CD8 Microbeads (Miltenyi Biotec, Cologne, Germany) at day 8. Splenocytes pulsed with 1 µM peptides or CD8 T cell-depleted lung cells were added as APCs and cultured with AMs at a ratio of 10∶1 for 72 h. Cells were then harvested, stained with antibodies and subjected to flow cytometric analysis.

### Purification of lung DCs

aDC population were purified from the lungs of naïve BALB/c mice by FACS sorting based on their expression of CD11c^+^MHC II^+^CD11b^−^ (Fig. S1) and enriched to about 80% purity.

### Generation of BM-derived DCs and adoptive transfer

Bone marrow-derived DCs (BMDC) were generated as previously described [56]. Briefly, red blood cell-depleted BM cells were plated at a density of 1×10^6^/ml in RP10 (RPMI with 10% fetal calf serum, 1.0 mM HEPES, 0.2 mM L-glutamine, 0.05 mM gentamicin sulfate, 1% penicillin- streptomycin, 1 mM sodium pyruvate, and 0.02 mM 2-mercaptoethanol) supplemented with 1,000 U/ml recombinant granulocyte-macrophage colony stimulating factor (BD Pharmingen) and 50 U/ml recombinant interleukin-4 (eBioscience). Cells were incubated for 6 days, with 75% medium replacement every 2 days. At day 6, BMDCs were stimulated with or without 20 µg/ml Poly I:C or 1 µg/ml LPS for 18–24 h. CD11c microbeads and a Miltenyi autoMACS magnetic cell sorter (Miltenyi Biotec, Cologne, Germany) were used to purify CD11c^+^ DCs. Purity was confirmed by flow cytometry. BALB/c mice were lightly anesthetized with isoflurane and 3×10^5^ BMDCs in 75 µl PBS were adoptively transfer i.n. 18 h before MA15 infection.

### Statistical analysis

A Student's *t* test was used to analyze differences in mean values between groups. All results are expressed as means±standard errors of the means (SEM). *P* values of <0.05 were considered statistically significant.

## Supporting Information

Figure S1 Gating strategy for DC and AM. (A) Gating strategy for aDCs, iDCs and AMs. Lungs were harvested, digested with collagenase, and examined for aDCs, iDCs and AMs populations by flow cytometry gating on the following markers: iDCs, CD11c^+^CD11b^+^MHC II^+^; aDCs, CD11c^+^CD11b^−^MHC II^+^; AM, CD11c^+^CD11b^−^Siglec F^+^. (B) Gating strategy for migratory DCs. Mice were treated with 50 µl 8 mM CFSE i.n. 6 h after CFSE instillation, single cell suspensions were prepared from lung DLNs and gated for CD11c expression by flow cytometry. Representative side scatter versus CFSE staining of CD11c^+^ gated cells is shown.
(2.30 MB TIF)

Figure S2 MA15 infection did not activate AM *in vitro*. AMs were harvested from BAL fluid and infected with MA15 (multiplicity of infection = 5) for 24 h Expression of CD86 were determined by flow cytometry. Data are representative of three independent experiments.
(0.42 MB TIF)

Figure S3 Depletion of alveolar macrophages by clodronate-liposomes. Mice were treated with 75 µl clodronate-liposomes, or PBS i.n. 24 or 48 h after treatment, lungs were examined by flow cytometry for frequency (A) and total numbers (B) of AM (CD11c^+^CD11b^−^SiglecF^+^). Data are representative of four independent experiments and are the mean values±SEM (*n* = 8 mice/group/time point).
(0.71 MB TIF)

Figure S4 Activation of T cells during MA15 infection. Mice treated with CL or PBS were infected with 3×10^4^ PFU MA15 virus. At the indicated time points, single cell suspension were prepared from lungs and the expression of CD43 (mAb 1B11), CD8 and CD4 determined by flow cytometry. Frequency and numbers of CD8 (A) and CD4 (B) T cells are shown. Data are representative of two independent experiments and are the mean values±SEM (*n* = 6–8 mice/group/time point).
(1.58 MB TIF)

Figure S5 F4/80 and CD200R expression on alveolar and peritoneal macrophages. AMs were harvested from BAL fluid. To obtain peritoneal macrophages, mice were inoculated with 2 ml 3% thioglycolate media 4 days before peritoneal lavage. Cells were examined by flow cytometry for expression of F4/80 and CD200R (solid line). Gray, isotype control. Change of Mean fluorescence intensity (ΔMFI) = MFI_test_−MFI_iso_. ΔMFI of F4/80 expression: PM (87.4) vs AM (13.4), ΔMFI of CD200R expression: PM (39.3) vs AM (115). Data are representative of three independent experiments.
(0.48 MB TIF)

Figure S6 Cytokine expression after AM and T cell co-culture and requirement for direct AM-T cell contact for inhibition of cell proliferation. (A) AMs were harvested from bronchoalveolar lavage fluid (BALF) and cultured at 4×10^4^ in each 96-well. AM-depleted MA15-infected lung cells were stimulated with SARS-CoV CD8 (S366, S521 and S1061) peptides for 6 h in the presence or absence AMs. Brefeldin A was added during the last 2 h of co-culture. IFN-γ expression was determined by intracellular staining. Data are representative of three independent experiments. (B) AMs were harvested from BAL fluid and cultured at 2.5×10^5^ /well in 24-well dishes for 48 h before use. Single cell suspension were prepared from spleens of naïve mice, stained with 1 µM CFSE, stimulated with either 2.5 µg/ml Con A or 1 µg/ml soluble CD3 antibody for 72 h above a semi-membrane, and subjected to flow cytometry. Data are representative of two independent experiments.
(0.85 MB TIF)

Figure S7 rDC phenotypic changes after poly I:C and LPS treatment *in vivo*. Mice were treated with 20 µg poly I:C or 5 µg LPS for 18–24 h. Single cell suspension were prepared from lungs. CD86 and CD40 expression on aDCs (CD11c^+^CD11b^−^MHC II^+^) and iDCs (CD11c^+^CD11b^+^MHC II^+^) were determined by flow cytometry. The frequencies of MHC II^high^CD86^+^ or CD40^+^MHC II^high^ populations are shown. Data are representative of three independent experiments.
(1.19 MB TIF)

Table S1 Bio-plex assay for cytokines and chemokines production during MA15 infection. Mice were treated with either PBS or CL 24 h before MA15 infection. Lungs were harvested at day 0, day 2 and day 4 p.i. After homogenization and ultraviolet light inactivation, samples were analyzed for cytokine and chemokine expression using a Bio-Plex cytometric bead assay and a Luminex 200 luminometer (Bio-Rad). The concentration of cytokines and chemockines was expressed as pg/ml lung homogenate. *P values of <0.05.
(0.04 MB DOC)
