# Supplementary figures and images for: Evasion by Stealth: Inefficient Immune Activation Underlies Poor T Cell Response and Severe Disease in SARS-CoV-Infected Mice
Source: PLoS Pathog. 2009 Oct 23;5(10):e1000636. doi: 10.1371/journal.ppat.1000636 (PMC2762542; doi:10.1371/journal.ppat.1000636)

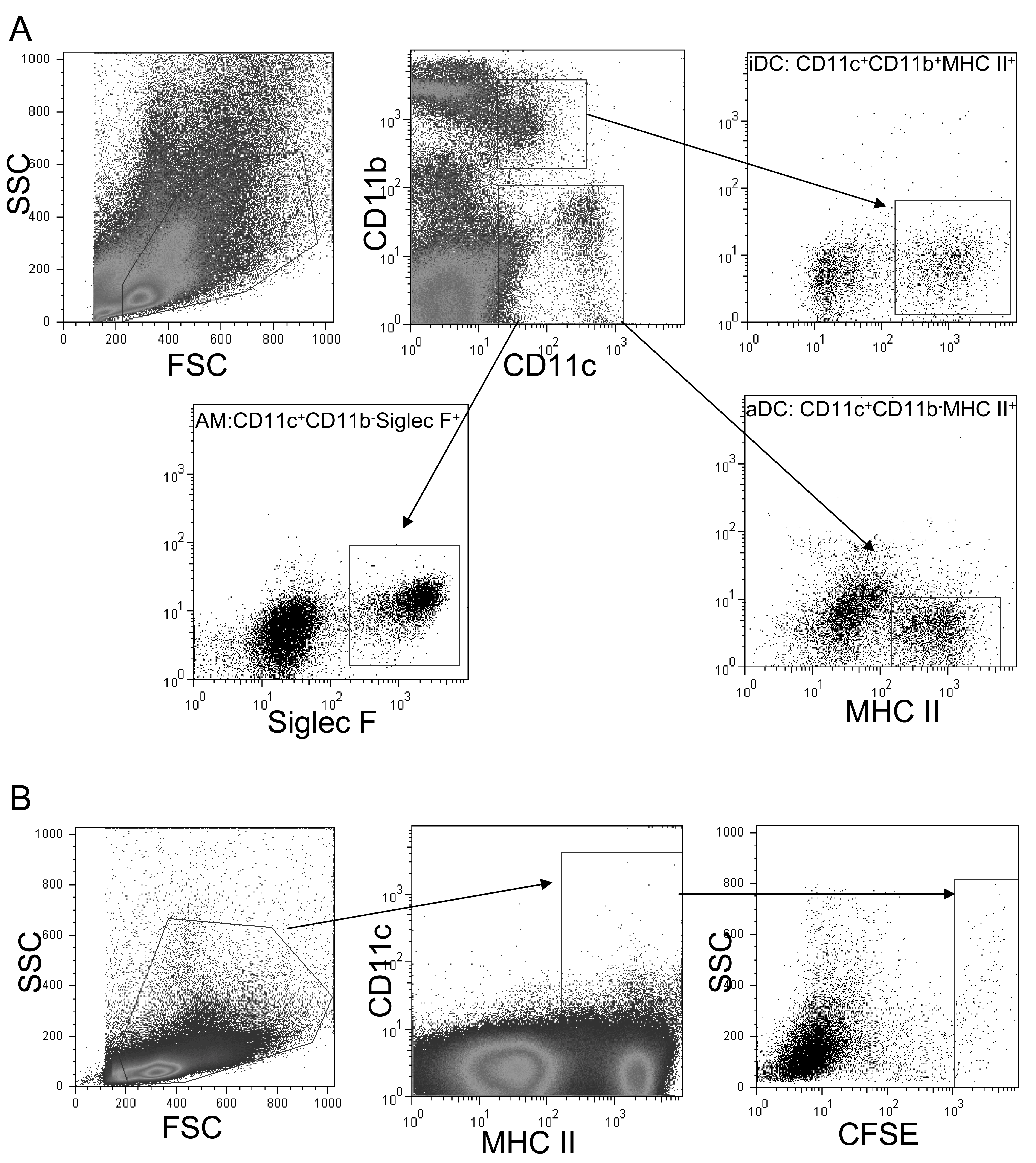

Supplement: Figure S1 — Gating strategy for DC and AM. (A) Gating strategy for aDCs, iDCs and AMs. Lungs were harvested, digested with collagenase, and examined for aDCs, iDCs and AMs populations by flow cytometry gating on the following markers: iDCs, CD11c+CD11b+MHC II+; aDCs, CD11c+CD11b−MHC II+; AM, CD11c+CD11b−Siglec F+. (B) Gating strategy for migratory DCs. Mice were treated with 50 µl 8 mM CFSE i.n. 6 h after CFSE instillation, single cell suspensions were prepared from lung DLNs and gated for CD11c expression by flow cytometry. Representative side scatter versus CFSE staining of CD11c+ gated cells is shown. (2.30 MB TIF) [file ppat.1000636.s001.tif]

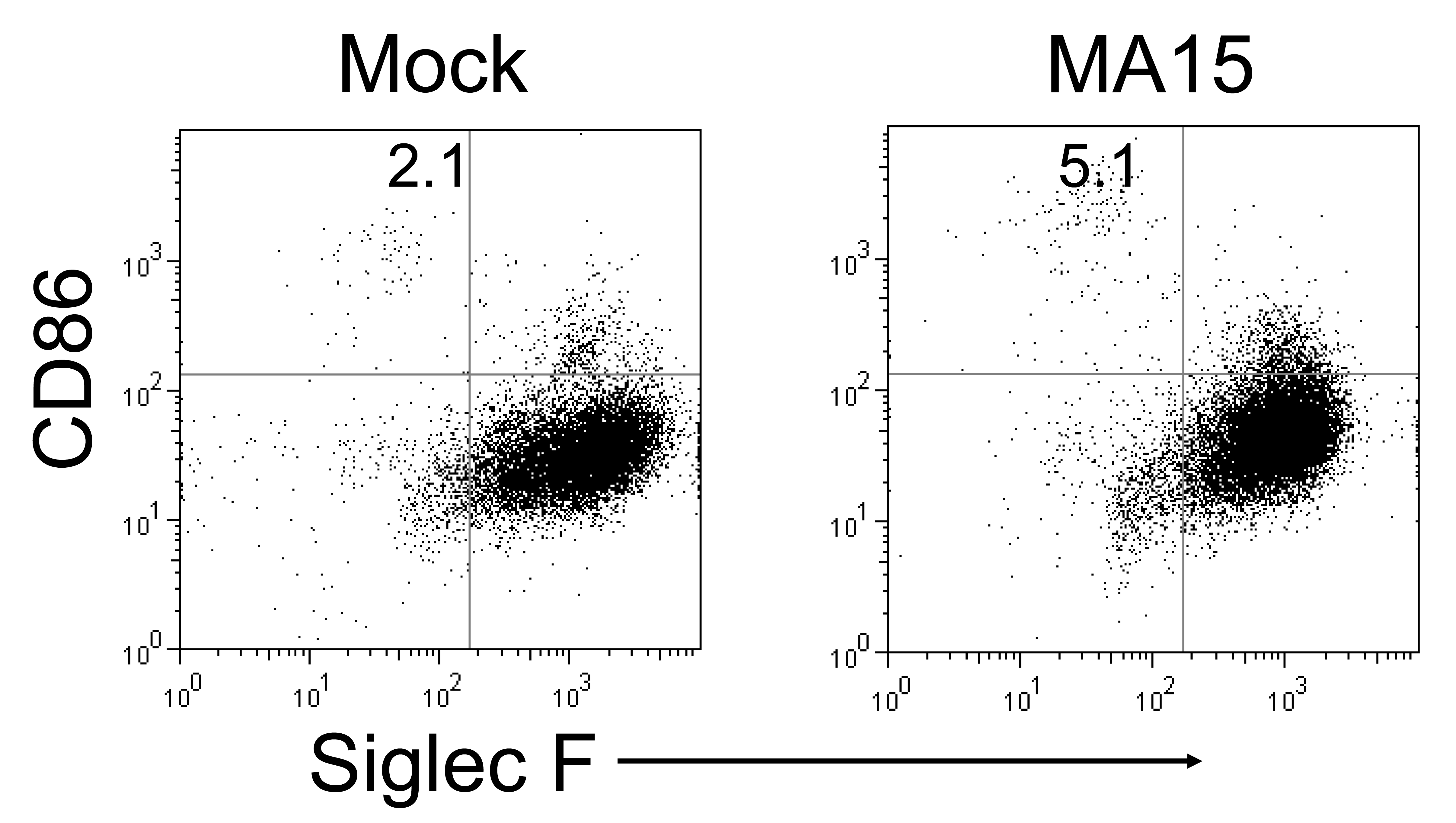

Supplement: Figure S2 — MA15 infection did not activate AM in vitro. AMs were harvested from BAL fluid and infected with MA15 (multiplicity of infection = 5) for 24 h Expression of CD86 were determined by flow cytometry. Data are representative of three independent experiments. (0.42 MB TIF) [file ppat.1000636.s002.tif]

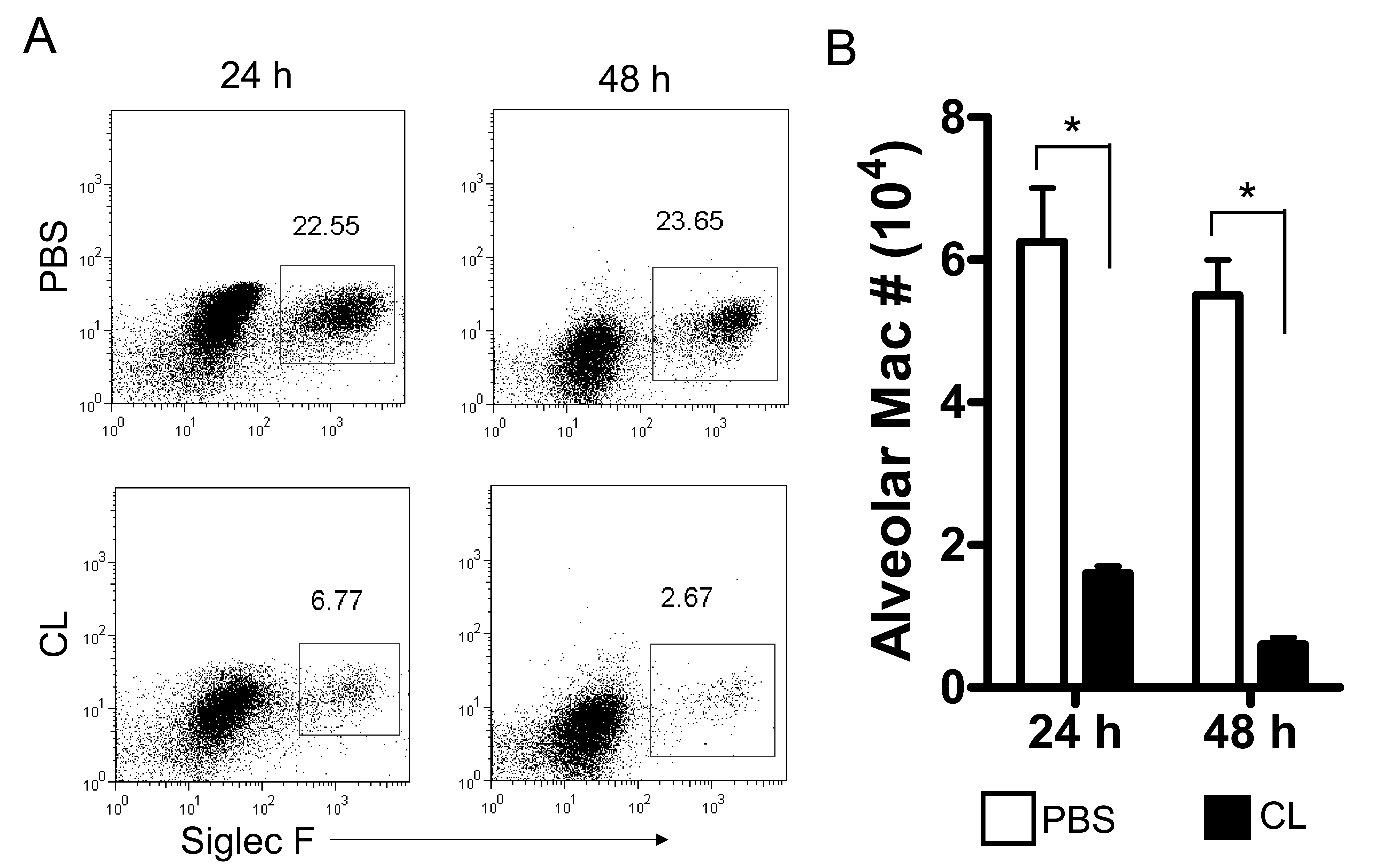

Supplement: Figure S3 — Depletion of alveolar macrophages by clodronate-liposomes. Mice were treated with 75 µl clodronate-liposomes, or PBS i.n. 24 or 48 h after treatment, lungs were examined by flow cytometry for frequency (A) and total numbers (B) of AM (CD11c+CD11b−SiglecF+). Data are representative of four independent experiments and are the mean values±SEM (n = 8 mice/group/time point). (0.71 MB TIF) [file ppat.1000636.s003.tif]

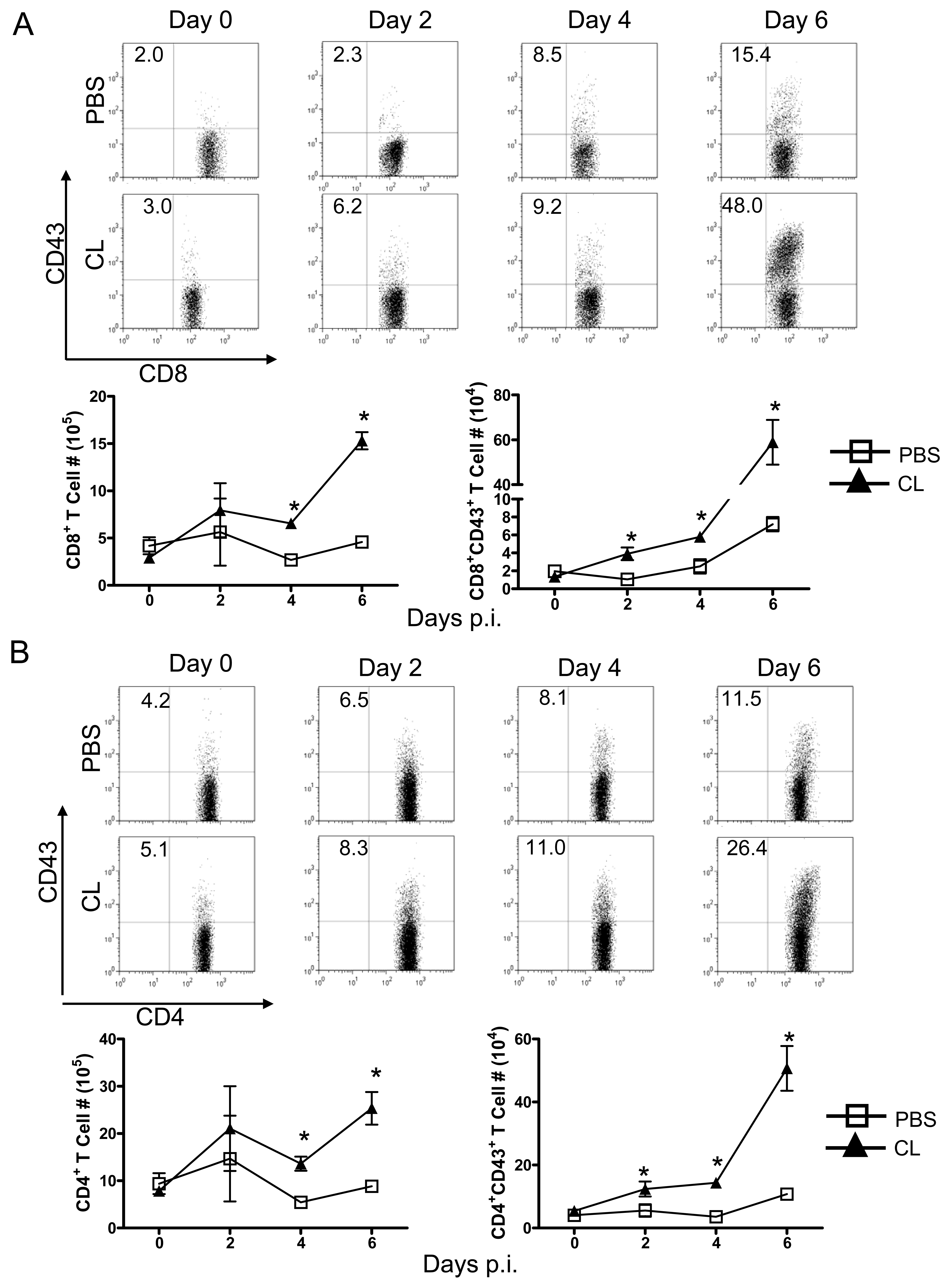

Supplement: Figure S4 — Activation of T cells during MA15 infection. Mice treated with CL or PBS were infected with 3×10^4^ PFU MA15 virus. At the indicated time points, single cell suspension were prepared from lungs and the expression of CD43 (mAb 1B11), CD8 and CD4 determined by flow cytometry. Frequency and numbers of CD8 (A) and CD4 (B) T cells are shown. Data are representative of two independent experiments and are the mean values±SEM (n = 6–8 mice/group/time point). (1.58 MB TIF) [file ppat.1000636.s004.tif]

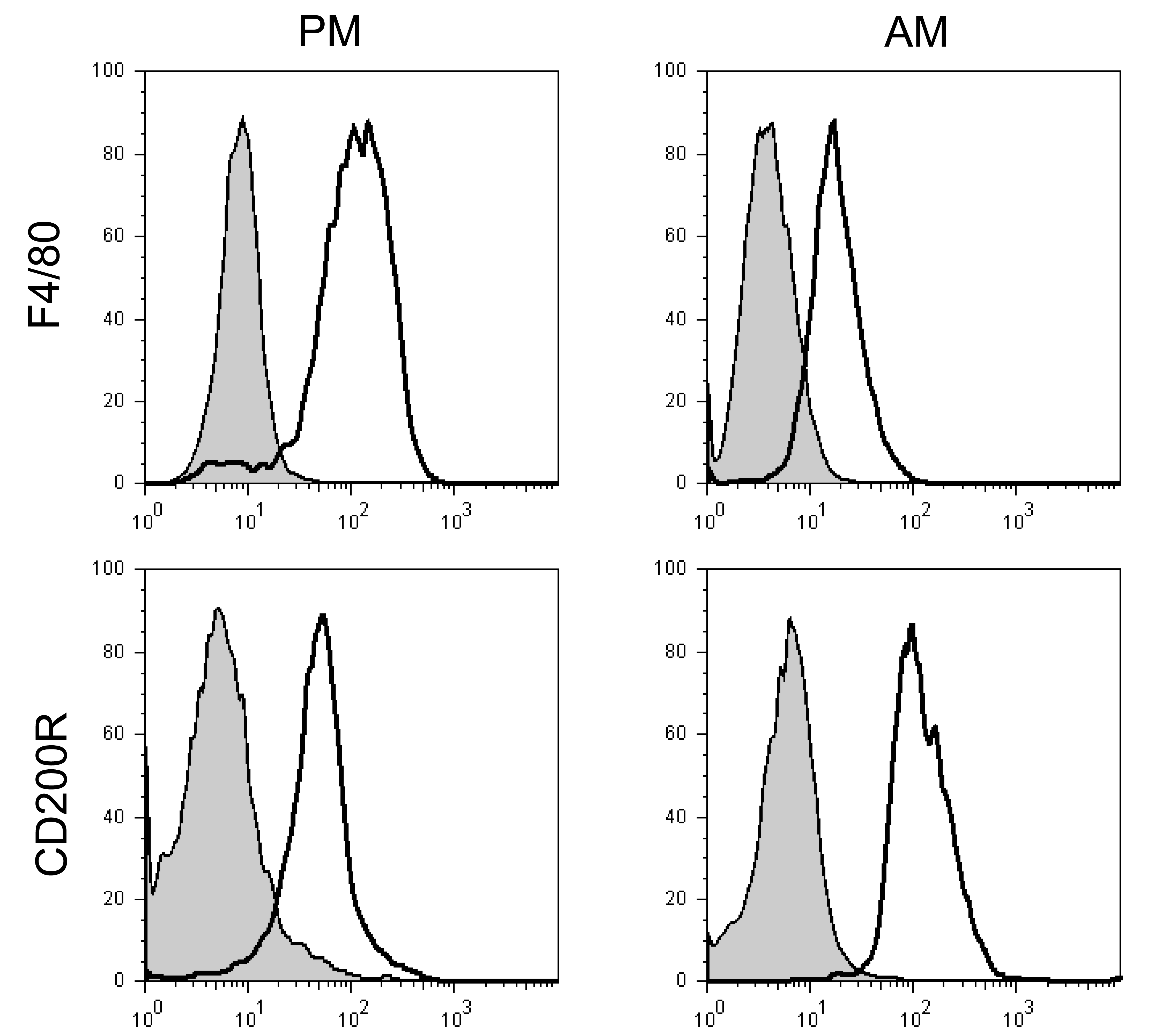

Supplement: Figure S5 — F4/80 and CD200R expression on alveolar and peritoneal macrophages. AMs were harvested from BAL fluid. To obtain peritoneal macrophages, mice were inoculated with 2 ml 3% thioglycolate media 4 days before peritoneal lavage. Cells were examined by flow cytometry for expression of F4/80 and CD200R (solid line). Gray, isotype control. Change of Mean fluorescence intensity (ΔMFI) = MFItest−MFIiso. ΔMFI of F4/80 expression: PM (87.4) vs AM (13.4), ΔMFI of CD200R expression: PM (39.3) vs AM (115). Data are representative of three independent experiments. (0.48 MB TIF) [file ppat.1000636.s005.tif]

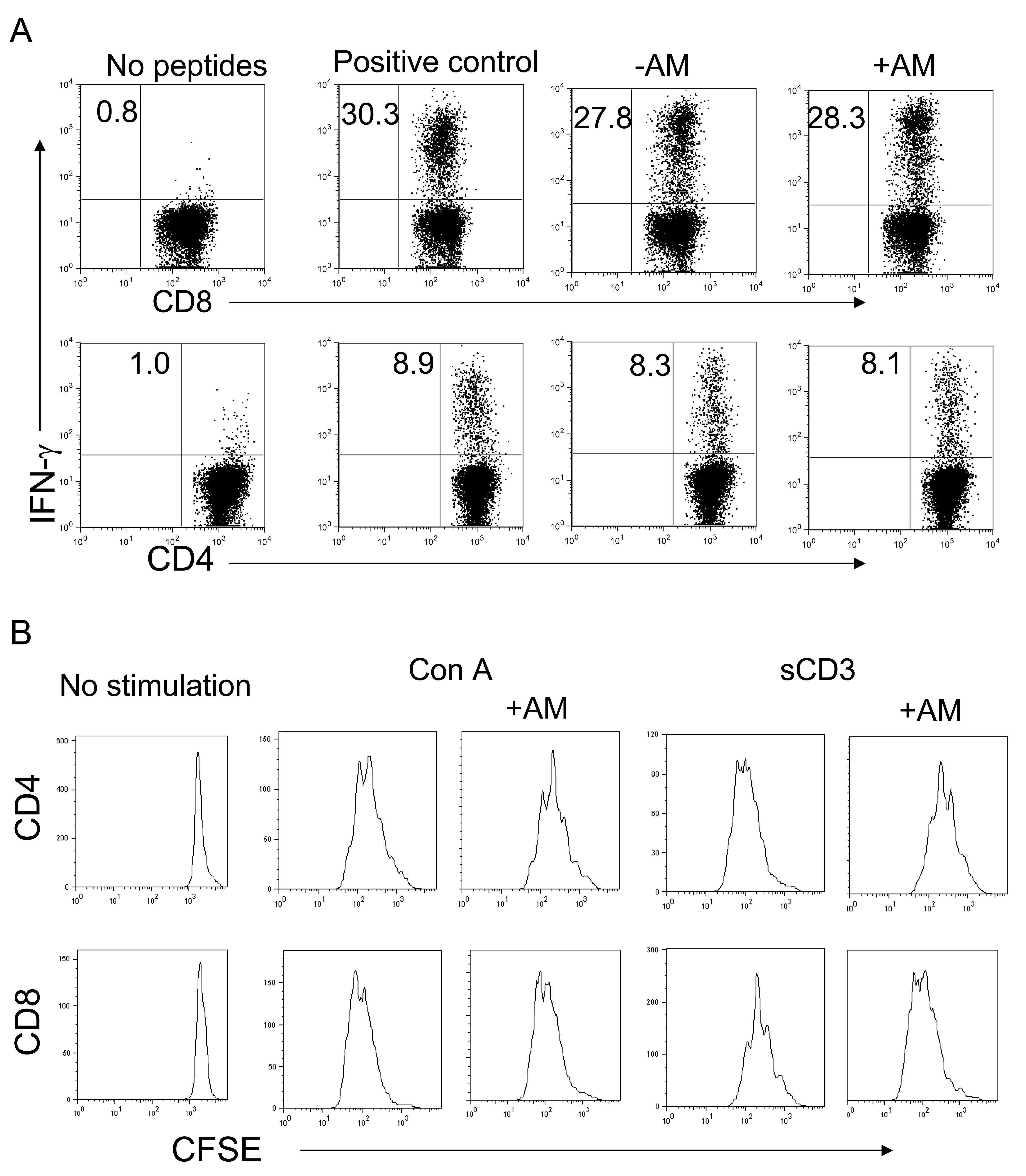

Supplement: Figure S6 — Cytokine expression after AM and T cell co-culture and requirement for direct AM-T cell contact for inhibition of cell proliferation. (A) AMs were harvested from bronchoalveolar lavage fluid (BALF) and cultured at 4×10^4^ in each 96-well. AM-depleted MA15-infected lung cells were stimulated with SARS-CoV CD8 (S366, S521 and S1061) peptides for 6 h in the presence or absence AMs. Brefeldin A was added during the last 2 h of co-culture. IFN-γ expression was determined by intracellular staining. Data are representative of three independent experiments. (B) AMs were harvested from BAL fluid and cultured at 2.5×10^5^ /well in 24-well dishes for 48 h before use. Single cell suspension were prepared from spleens of naïve mice, stained with 1 µM CFSE, stimulated with either 2.5 µg/ml Con A or 1 µg/ml soluble CD3 antibody for 72 h above a semi-membrane, and subjected to flow cytometry. Data are representative of two independent experiments. (0.85 MB TIF) [file ppat.1000636.s006.tif]

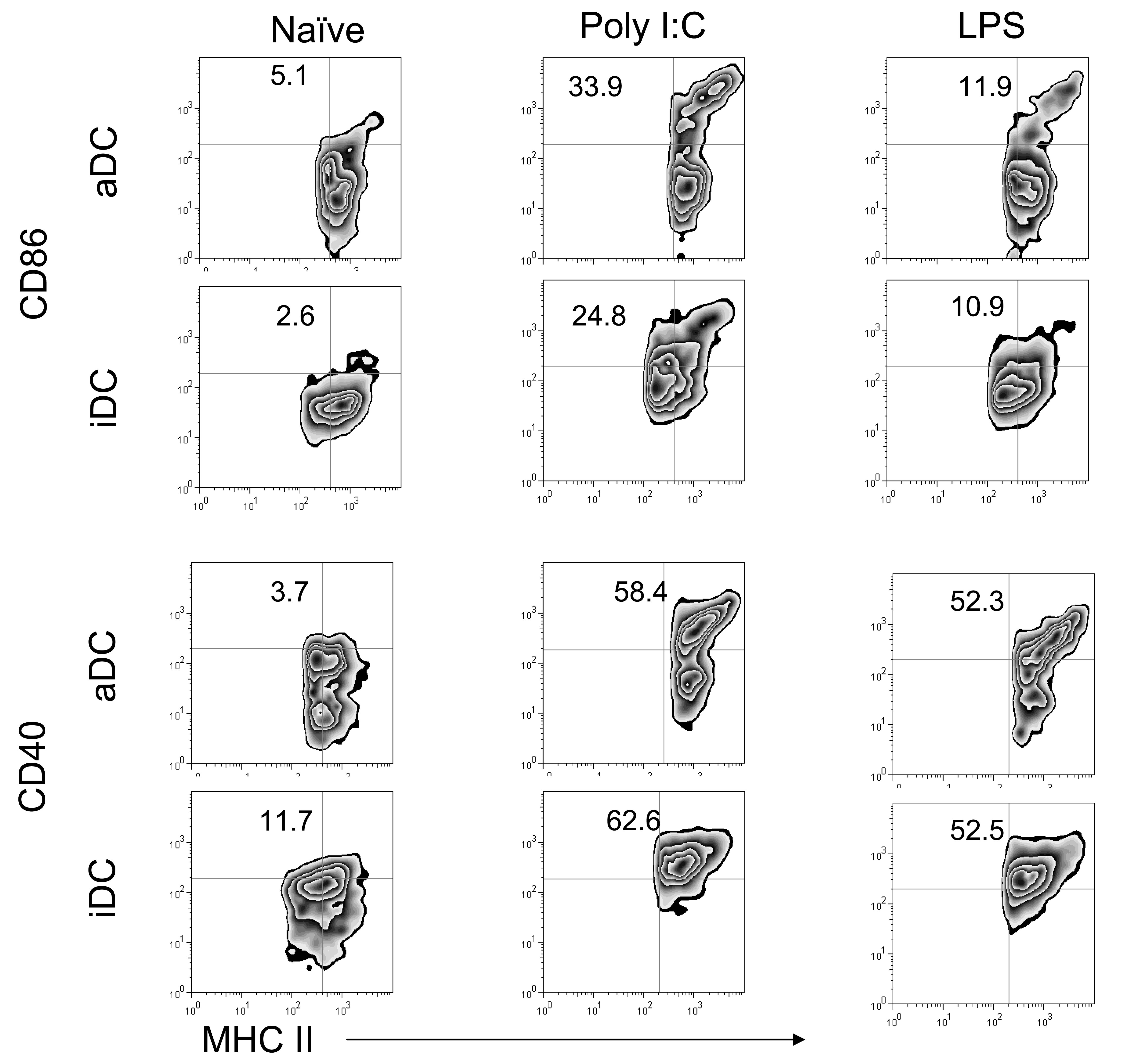

Supplement: Figure S7 — rDC phenotypic changes after poly I:C and LPS treatment in vivo. Mice were treated with 20 µg poly I:C or 5 µg LPS for 18–24 h. Single cell suspension were prepared from lungs. CD86 and CD40 expression on aDCs (CD11c+CD11b−MHC II+) and iDCs (CD11c+CD11b+MHC II+) were determined by flow cytometry. The frequencies of MHC IIhighCD86+ or CD40+MHC IIhigh populations are shown. Data are representative of three independent experiments. (1.19 MB TIF) [file ppat.1000636.s007.tif]
