# Supplementary material for: Evasion by Stealth: Inefficient Immune Activation Underlies Poor T Cell Response and Severe Disease in SARS-CoV-Infected Mice
Source: PLoS Pathog. 2009 Oct 23;5(10):e1000636. doi: 10.1371/journal.ppat.1000636 (PMC2762542; doi:10.1371/journal.ppat.1000636)
Supplement: Table S1 — Bio-plex assay for cytokines and chemokines production during MA15 infection. Mice were treated with either PBS or CL 24 h before MA15 infection. Lungs were harvested at day 0, day 2 and day 4 p.i. After homogenization and ultraviolet light inactivation, samples were analyzed for cytokine and chemokine expression using a Bio-Plex cytometric bead assay and a Luminex 200 luminometer (Bio-Rad). The concentration of cytokines and chemockines was expressed as pg/ml lung homogenate. *P values of <0.05. (0.04 MB DOC) [file ppat.1000636.s008.doc]

**Table S1. Bio-plex assay for cytokines and chemokines production during MA15 infection.**

|  | **Day 0** | | | **Day 2** | | **Day 4** | |
| --- | --- | --- | --- | --- | --- | --- | --- |
| pg/ml | **PBS** | **CL** | | **PBS** | **CL** | **PBS** | **CL** |
| **G-CSF** | *17±3 | | 305±100 | 514±467 | 358±133 | 1072±802 | 573±307 |
| **GM-CSF** | *26±4 | | 354±153 | 44±12 | 59±9 | 53±18 | 70±12 |
| **IL-1α** | 32±3 | | 93±38 | *167±28 | 57±6 | 31±10 | 34±7 |
| **IL-1β** | *83±41 | | 896±336 | 864±289 | 567±119 | 438±168 | 580±82 |
| **IL-2** | *37±1 | | 95±18 | 11±10 | 15±13 | 17±11 | 18±4 |
| **IL-6** | *35±0 | | 145±22 | 145±35 | 63±3 | 77±64 | 43±12 |
| **IL-12 (P40)** | *45±20 | | 362±127 | 2008±1062 | 3378±887 | 1578±895 | 3212±581 |
| **MCP-1** | *335±51 | | 1323±169 | 122459±139276 | 58516±43547 | 31373±30254 | 21917±13407 |
| **MIP-1α** | *158±33 | | 1114±111 | 3562±1282 | 3834±731 | 2076±1143 | 4127±264 |
| **MIP-1β** | *53±5 | | 223±59 | 630±210 | 619±41 | 422±150 | 600±82 |
